# Exploring novel and potent cell penetrating peptides in the proteome of SARS-COV-2 using bioinformatics approaches

**DOI:** 10.1371/journal.pone.0247396

**Published:** 2021-02-19

**Authors:** Kimia Kardani, Azam Bolhassani

**Affiliations:** Department of Hepatitis and AIDS, Pasteur Institute of Iran, Tehran, Iran; Nanyang Technological University, SINGAPORE

## Abstract

Among various delivery systems for vaccine and drug delivery, cell-penetrating peptides (CPPs) have been known as a potent delivery system because of their capability to penetrate cell membranes and deliver some types of cargoes into cells. Several CPPs were found in the proteome of viruses such as Tat originated from human immunodeficiency virus-1 (HIV-1), and VP22 derived from herpes simplex virus-1 (HSV-1). In the current study, a wide-range of CPPs was identified in the proteome of SARS-CoV-2, a new member of coronaviruses family, using *in silico* analyses. These CPPs may play a main role for high penetration of virus into cells and infection of host. At first, we submitted the proteome of SARS-CoV-2 to CellPPD web server that resulted in a huge number of CPPs with ten residues in length. Afterward, we submitted the predicted CPPs to C2Pred web server for evaluation of the probability of each peptide. Then, the uptake efficiency of each peptide was investigated using CPPred-RF and MLCPP web servers. Next, the physicochemical properties of the predicted CPPs including net charge, theoretical isoelectric point (pI), amphipathicity, molecular weight, and water solubility were calculated using protparam and pepcalc tools. In addition, the probability of membrane binding potential and cellular localization of each CPP were estimated by Boman index using APD3 web server, D factor, and TMHMM web server. On the other hand, the immunogenicity, toxicity, allergenicity, hemolytic potency, and half-life of CPPs were predicted using various web servers. Finally, the tertiary structure and the helical wheel projection of some CPPs were predicted by PEP-FOLD3 and Heliquest web servers, respectively. These CPPs were divided into: a) CPP containing tumor homing motif (RGD) and/or tumor penetrating motif (RXXR); b) CPP with the highest Boman index; c) CPP with high half-life (~100 hour) in mammalian cells, and d) CPP with +5.00 net charge. Based on the results, we found a large number of novel CPPs with various features. Some of these CPPs possess tumor-specific motifs which can be evaluated in cancer therapy. Furthermore, the novel and potent CPPs derived from SARS-CoV-2 may be used alone or conjugated to some sequences such as nuclear localization sequence (NLS) for vaccine and drug delivery.

## Introduction

Therapeutic and preventive vaccines are promising approaches to solve health issues globally [1]. Although there are several vaccines for saving millions of lives till now such as vaccines against rubella, mumps, varicella, rotavirus, human papillomavirus (HPV) and hepatitis B virus (HBV), it is required to develop effective vaccines against other pathogens which are incurable and unprotectable [2,3]. In this line, development of effective and novel delivery systems is vital for delivery of vaccine components into cells. In general, delivery systems can be used to transfer different biomolecules into cells including nanoparticles [4], polymers [5], chitosan [6], liposome [7], physical tools [8], and cell penetrating peptides (CPPs) [9,10]. The current focus of developing a novel delivery system has moved to peptide-based delivery systems known as CPPs [11]. CPPs contain 5–50 amino acids in length which can enter cell membranes efficiently and deliver a wide range of cargoes including peptides, proteins, nanoparticles and nucleic acids into cells [12,13]. After discovery of the first CPP, Tat peptide (originated from human immunodeficiency virus type-1 (HIV-1) trans-activating regulatory (Tat) protein), a rapid growth of new CPPs has occurred [14]. The CPPs are natural (*e*.*g*., CyLoP-1) or synthetic (*e*.*g*., oligoarginine) peptides. These short peptides are heterogeneous in sequence and structure, and can be delivered through endocytosis or direct penetration [1,10,15–17]. The mechanism of internalization depends on diverse factors such as CPP sequence, cell type, CPP concentration, temperature, incubation time, and type of cargo [18]. Up to now, a large number of CPPs have been recognized but some of them showed low uptake [19]. The studies demonstrated that prediction of CPPs by bioinformatics tools prior to lab-based experiments could save time and money [20]. For instance, machine-learning-based algorithms permit users to predict CPPs from large sequence data/ proteome. In prediction methods, machine learning models utilize various algorithms including neural network (NN) [21,22], kernel extreme learning machine [23,24], random forest (RF) [25], and support vector machine (SVM) [26,27].

In December 2019, a new member of the coronavirus family was found which firstly named as 2019-nCoV. Then, on February 11, 2020, its name was changed to Coronavirus Disease-2019 (COVID-19) or severe acute respiratory syndrome coronavirus-2 (SARS-CoV-2) [28]. SARS-CoV-2 is an enveloped positive single-strand RNA virus that has 10 open reading frames (ORFs). ORF1ab is about 66.66% of virus genome which encodes two large polypeptides such as pp1a and pp1ab. Meanwhile, the ORF2-10 is about 33.33% of virus genome. SARS-CoV-2 genome encodes 28 proteins which are classified into three various classes such as structural proteins, non-structural proteins (nsp), and accessory proteins. The structural proteins are spike (S), nucleoprotein (N), membrane (M), and envelope (E) proteins that form the virus particles. In addition, the non-structural proteins (*e*.*g*., nsp1-nsp16) are generated only during the translation of virus RNA in the infected host cell. The accessory proteins possess crucial functions in the assembly, virulence and pathogenesis of the virus [28,29].

Previously, several CPPs were derived from viruses such as Tat (from HIV-1 transcriptional activator protein), C105Y (from HIV-1 glycoprotein 41), MPG (from HIV-1 glycoprotein 41 conjugated to nuclear localization sequence (NLS) from simian virus 40 (SV40)), Pep-1 (from HIV-1 reverse transcriptase conjugated to SV40 NLS), pepR and pepM (originated from Dengue virus), VP22 (originated from Herpes simplex virus (HSV)-1) [14,30–34]. Up to now, no complete report has been available on CPPs derived from total proteins of MERS-CoV, SARS-CoV and SARS-CoV-2. Few studies indicated that some peptides of SARS-CoV spike glycoprotein are responsible for membrane fusion or membrane binding activity. For example, the upstream region of the heptad repeat1 (HR1) (residues 892–972) in S2 domain of SARS-CoV spike glycoprotein was involved in membrane fusion. Moreover, some scientists have recognized membrane binding peptides and membrane fusogenic peptides or potential fusion peptides from the upstream region of HR1 (residues 758–890) [35]. Indeed, an efficient membrane fusion mechanism between host cell and SARS-CoV-2 can be responsible for virus infection. Sequence comparison of S protein domains between SARS-CoV-2 and SARS-CoV-1 showed high level of conservation for both S1 and S2 domains. However, variation in the fusogenic regions of S2 domain was observed between SARS-CoV-2 and SARS-CoV-1 [36–38]. Hence, due to high potency of SARS-CoV-2 to spread and infect people, we decided to investigate new and potent CPPs in the proteome of this newly isolated virus using *in silico* approaches.

## Materials and methods

### Study design

The current study has several main steps to find and characterize novel and potent CPPs as a vaccine and drug delivery system. The flowchart of overall prediction and analysis procedure was illustrated in **S1 Fig.**

### Identification of potential SARS-CoV-2-derived CPPs

Cell penetrating or non-cell penetrating peptides (CPP or non-CPP) could be predicted in the proteome of SARS-CoV-2 using bioinformatics approaches. Hence, to explore novel CPPs, our reference sequence was Wuhan-Hu-1 with GenBank accession number MN908947.3. This strain was isolated from a patient in Wuhan, china. The phylogenetic analysis of whole viral genome contain 29,903 nucleotides that has 89.1% nucleotide similarity to a group of SARS-like coronaviruses (genus Betacoronavirus, subgenus Sarbecovirus) which formerly had been recognized in bats [39].

At first, CellPPD web server (https://webs.iiitd.edu.in/raghava/cellppd/index.html) was applied to determine the cell penetrating peptides. CellPPD is a support vector machine (SVM)-based web server [40,41]. To utilize this web server, the sequences of Spike (S) protein (GenBank ID: QHD43416.1), Membrane (M) glycoprotein (GenBank ID: QHD43419.1), Nucleocapsid (N) phosphoprotein (GenBank ID: QHD43423.2), Envelope (E) protein (GenBank ID: QHD43418.1), Orf1ab polyprotein (GenBank ID: QHD43415.1), ORF3a protein (GenBank ID: QHD43417.1), ORF6 protein (GenBank ID: QHD43420.1), ORF7a protein (GenBank ID: QHD43421.1), ORF8 protein (GenBank ID: QHD43422.1), and ORF10 protein (GenBank ID: QHI42199.1) were submitted to protein scanning tool with default threshold of the SVM-based prediction method (SVM threshold was set at 0.0). Moreover, Tang and colleagues [42] developed a method with an overall prediction accuracy of 83.6%; hence, they established C2Pred web server (http://lin-group.cn/server/C2Pred) to investigate the CPP probability of each peptide.

### Uptake efficiency analysis of the identified CPPs

In next step, to evaluate the uptake efficiency of the identified CPPs from previous step, two web servers including CPPred-RF (http://server.malab.cn/CPPred-RF/), and MLCPP (http://www.thegleelab.org/MLCPP/) were used. For this purpose, all of the detected CPPs using CellPPD web server were submitted to these web servers. CPPred-RF is a sequence-based predictor for identifying CPPs and their uptake efficiency. In addition, CPPred-RF built a two-layer prediction framework according to the random forest (RF) algorithm [43]. Manavalan *et al*. established a two-layer prediction framework termed as machine-learning-based prediction of cell-penetrating peptide (MLCPPs). The first-layer predicts that a submitted peptide is categorized as a CPP or non-CPP. Meanwhile, the second-layer predicts the uptake efficiency of the predicted CPPs [44].

### Peptides property calculation

It is crucial to compute the physicochemical properties of peptides for predicting and designing novel and potent CPPs. Therefore, to achieve this aim, we calculated various physicochemical features of CPPs such as net charge, theoretical isoelectric point (pI), amphipathicity, molecular weight (MW), water solubility, hydrophobicity (H), hydrophobicity ratio, and polar-, non-polar-, uncharged- and charged residues. To calculate net charge, theoretical pI, and amphipathicity, CellPPD web server (https://webs.iiitd.edu.in/raghava/cellppd/index.html) was utilized. In addition, protparam tool (https://web.expasy.org/protparam/) was used to compute molecular weight of CPPs. Furthermore, to obtain the water solubility of peptides, Peptide property calculator (PepCalc) (https://pepcalc.com/) was applied. Also, hydrophobicity (H), and polar-, non-polar-, uncharged- and charged residues were estimated using Heliquest web server (https://heliquest.ipmc.cnrs.fr/cgi-bin/ComputParams.py).

### Evaluation of membrane-binding ability of CPPs

In order to investigate the potential of binding peptides to membrane, two different methods were utilized. At first, we evaluated the Boman index or protein-binding potential using APD3 web server (http://aps.unmc.edu/AP/prediction/prediction_main.php). The Boman index is the sum of solubility values for all presented amino acids in a peptide sequence and illustrates the potential of a peptide for binding to the membrane or other proteins [45]. Secondly, to evaluate the membrane-binding potential of each peptide, the discrimination factor (D) was calculated [46]. For this purpose, we used Heliquest web server (https://heliquest.ipmc.cnrs.fr/cgi-bin/ComputParams.py) to obtain hydrophobic moment (μH). After determination of hydrophobic moment and also net charge (Z), the D factor was calculated according to the following equation: D = 0.944(<μH>) + 0.33(Z).

In addition, TMHMM web server (http://www.cbs.dtu.dk/services/TMHMM/) was utilized to investigate the cellular localization of CPPs [47]. This web server analyzes the probability of binding a peptide to the bacterial cell membrane (BCM) which possesses negative charge.

### Assessment of the immunogenicity

Immunogenicity of the CPPs is one of their disadvantages. It was confirmed that peptides could induce immunologic responses *in vivo*, resulting in allergic reactions. The existence of peptides in body can stimulate the generation of antibodies which may neutralize therapeutic effects and reduce their efficacy [48, 49]. Hence, to assess the immunogenicity of CPPs, each peptide was submitted to IEDB Immunogenicity Predictor (http://tools.iedb.org/immunogenicity/) [50].

### Determination of toxicity and allergenicity

To investigate the toxicity and allergenicity of CPPs, each peptide was submitted to ToxinPred web server (https://webs.iiitd.edu.in/raghava/toxinpred/algo.php), and AllerTop (https://www.ddg-pharmfac.net/AllerTOP/) and AllergenFP (http://ddg-pharmfac.net/AllergenFP/) web servers, respectively [51–53].

### Estimation of hemolytic potency and half-life

The hemolytic property of peptides was predicted by HemoPI using SVM-based method (https://webs.iiitd.edu.in/raghava/hemopi/design.php). Furthermore, the half-life in *E*.*coli* and in mammalian cell was calculated using ProtLifePred web server based on N-end rule (http://protein-n-end-rule.leadhoster.com/) [54].

### Prediction of structure

Three dimensional (3D) structure of some predicted CPPs was analyzed by *de novo* peptide structure prediction server (PEP-FOLD3) (https://bioserv.rpbs.univ-paris-diderot.fr/services/PEP-FOLD3/). PEP-FOLD3 is a *de novo* method that predicts peptide structures using amino acid sequences. This approach determines the conformation of four consecutive amino acid residues according to structural alphabet (SA) letters [55]. Additionally, the helical wheel diagram of CPPs was defined by Schiffer Edmundson wheel modelling using Heliquest web server (https://heliquest.ipmc.cnrs.fr/cgi-bin/ComputParams.py) [46].

## Results

### Identification of potential SARS-CoV-2-derived CPPs

To obtain cell penetrating peptides in the proteome of SARS-CoV-2, the sequences of S protein, M glycoprotein, N phosphoprotein, E protein, Orf1ab polyprotein, and ORF3a, ORF6, ORF7a, ORF8 and ORF10 proteins were submitted to protein scanning tools of CellPPD web server. Then, we applied C2Pred web server to achieve the CPP probability of peptides. All of the detected CPPs, and their SVM scores and probability scores were listed in **Table 1**. No CPP was found in E protein, and only one CPP was identified in ORF6. Meanwhile, Orf1ab had the most CPPs in its proteome. C2pred web server identifies peptides lower than 0.5 as non-CPPs, and peptides greater than 0.5 as CPPs. Although, some peptides were predicted as CPPs by CellPPD, but C2Pred detected them as non-CPPs. For instance, DMSKFPLKLR peptide derived from Orf1ab polyprotein was predicted as CPP by CellPPD with SVM score of 0.11, while C2Pred determined this peptide as non-CPP with score 0.167688.

**Table 1:** Predicted CPPs and their uptake efficiency using various web servers.

Epitope	CellPPD (SVM score)	CPP Probability score by C2Pred*	Uptake efficiency by MLCPP	Uptake efficiency by CPPred-RF
**S protein**				
NLTTRTQLPP	0.03	0.549136	Low	High
RFQTLLALHR	0.11	0.924753	Low	High
YLQPRTFLLK	0.05	0.745663	Low	High
SVYAWNRKRI	0.12	0.542719	Low	High
YAWNRKRISN	0.21	0.581372	Low	High
AWNRKRISNC	0.10	0.581372	Low	High
WNRKRISNCV	0.07	0.422121	Low	High
RQIAPGQTGK	0.05	0.480859	Low	High
YNYLYRLFRK	0.10	0.434133	High	High
YLYRLFRKSN	0.17	0.323746	High	High
YRLFRKSNLK	0.18	0.651294	Low	High
RLFRKSNLKP	0.18	0.633622	Low	High
RKSNLKPFER	0.14	0.800554	Low	High
KKSTNLVKNK	0.33	0.588285	Low	High
KSTNLVKNKC	0.06	0.527724	Low	High
HADQLTPTWR	0.00	0.545188	Low	Non-CPP
YQTQTNSPRR	0.11	0.149319	Low	High
TQTNSPRRAR	0.11	0.143146	Low	High
TNSPRRARSV	0.16	0.142480	Low	High
NSPRRARSVA	0.14	0.108221	Low	High
PRRARSVASQ	0.17	0.236319	Low	High
KQIYKTPPIK	0.46	0.944703	Low	High
SQILPDPSKP	0.03	0.906582	Low	High
RLITGRLQSL	0.21	0.663600	Low	High
**M protein**				
NRNRFLYIIK	0.02	0.220554	Low	High
RNRFLYIIKL	0.18	0.114117	Low	High
YIIKLIFLWL	0.01	0.669640	Low	High
KLIFLWLLWP	0.31	0.709344	Low	High
FIASFRLFAR	0.10	0.421173	Low	High
ASFRLFARTR	0.20	0.594022	Low	High
SFRLFARTRS	0.06	0.553645	Low	High
FRLFARTRSM	0.21	0.385465	Low	High
RLFARTRSMW	0.28	0.383433	Low	High
FARTRSMWSF	0.04	0.463913	Low	High
HGTILTRPLL	0.03	0.430916	Low	High
GAVILRGHLR	0.13	0.223997	High	High
RIAGHHLGRC	0.02	0.243928	Low	High
YSRYRIGNYK	0.05	0.897477	Low	High
**N protein**				
PQNQRNAPRI	0.09	0.413323	Low	High
ERSGARSKQR	0.06	0.261718	Low	High
RSGARSKQRR	0.54	0.292041	Low	High
SGARSKQRRP	0.25	0.300551	Low	High
GARSKQRRPQ	0.40	0.250417	Low	High
ARSKQRRPQG	0.17	0.298689	Low	High
RSKQRRPQGL	0.36	0.192042	Low	High
SKQRRPQGLP	0.06	0.164110	Low	High
KQRRPQGLPN	0.18	0.206509	Low	High
RRPQGLPNNT	0.12	0.216779	Low	High
QIGYYRRATR	0.09	0.531651	Low	High
IGYYRRATRR	0.23	0.604819	Low	High
GYYRRATRRI	0.29	0.965070	Low	High
YYRRATRRIR	0.58	0.952696	Low	High
YRRATRRIRG	0.59	0.935039	Low	High
RRATRRIRGG	0.56	0.939794	Low	High
RATRRIRGGD	0.33	0.924855	Low	High
TRRIRGGDGK	0.10	0.369598	Low	High
RIRGGDGKMK	0.11	0.250269	Low	High
GKMKDLSPRW	0.02	0.735642	Low	High
SQASSRSSSR	0.05	0.677066	Low	High
ASSRSSSRSR	0.17	0.304528	Low	High
SSRSSSRSRN	0.09	0.304528	Low	High
SRSSSRSRNS	0.14	0.297448	Low	High
RSSSRSRNSS	0.12	0.432735	Low	High
SSSRSRNSSR	0.13	0.315140	Low	High
SSRSRNSSRN	0.10	0.315140	Low	High
RSRNSSRNST	0.04	0.359073	Low	High
GSSRGTSPAR	0.06	0.716403	Low	High
AALALLLLDR	0.14	0.898706	Low	High
ALALLLLDRL	0.05	0.895190	Low	High
KKSAAEASKK	0.33	0.730535	Low	High
KSAAEASKKP	0.13	0.669679	Low	High
SAAEASKKPR	0.06	0.669679	Low	High
AAEASKKPRQ	0.10	0.963042	Low	High
AEASKKPRQK	0.15	0.96509	Low	High
EASKKPRQKR	0.34	0.988105	Low	High
ASKKPRQKRT	0.28	0.947786	Low	High
SKKPRQKRTA	0.32	0.947786	Low	High
KKPRQKRTAT	0.47	0.947786	Low	High
KPRQKRTATK	0.42	0.941370	Low	High
PRQKRTATKA	0.23	0.936878	Low	High
RQKRTATKAY	0.13	0.936878	Low	High
RQGTDYKHWP	0.02	0.249058	Low	High
FPPTEPKKDK	0.04	0.937092	Low	Non-CPP
PPTEPKKDKK	0.12	0.954110	Low	High
PTEPKKDKKK	0.20	0.965728	Low	High
TEPKKDKKKK	0.51	0.972132	Low	High
EPKKDKKKKA	0.35	0.969009	Low	High
PKKDKKKKAD	0.28	0.736106	Low	High
KKDKKKKADE	0.26	0.275127	Low	High
TQALPQRQKK	0.11	0.378326	Low	High
ALPQRQKKQQ	0.17	0.715403	Low	High
**ORF3a**				
SASKIITLKK	0.07	0.666607	Low	High
ASKIITLKKR	0.19	0.975854	Low	High
SKIITLKKRW	0.31	0.977206	Low	High
KIITLKKRWQ	0.56	0.977206	Low	High
IITLKKRWQL	0.04	0.981679	Low	High
KKRWQLALSK	0.47	0.908996	Low	High
KRWQLALSKG	0.31	0.711236	Low	High
VRIIMRLWLC	0.11	0.512905	Low	High
RIIMRLWLCW	0.25	0.512905	Low	High
IIMRLWLCWK	0.14	0.584110	Low	High
IMRLWLCWKC	0.06	0.584110	High	High
MRLWLCWKCR	0.17	0.584110	High	High
RLWLCWKCRS	0.22	0.578205	High	High
LWLCWKCRSK	0.28	0.516957	High	High
WLCWKCRSKN	0.12	0.456487	High	High
LCWKCRSKNP	0.16	0.456487	High	High
CWKCRSKNPL	0.14	0.815386	High	High
KCRSKNPLLY	0.10	0.172543	Low	High
**Orf1ab**				
IKRSDARTAP	0.00	0.611515	Low	High
KRSDARTAPH	0.08	0.611515	Low	High
PVAYRKVLLR	0.05	0.395891	High	High
VAYRKVLLRK	0.07	0.461402	High	High
AYRKVLLRKN	0.13	0.873577	Low	High
YRKVLLRKNG	0.05	0.873577	Low	High
RKVLLRKNGN	0.14	0.892119	Low	High
KVLLRKNGNK	0.01	0.921012	Low	High
FEIKLAKKFD	0.09	0.877125	Low	High
KTIQPRVEKK	0.39	0.961264	Low	High
TIQPRVEKKK	0.13	0.954110	Low	High
IQPRVEKKKL	0.13	0.953833	Low	High
SGLKTILRKG	0.02	0.624757	Low	High
LKTILRKGGR	0.24	0.808425	Low	High
KTILRKGGRT	0.15	0.805867	Low	High
GNFKVTKGKA	0.03	0.385736	Low	High
FKVTKGKAKK	0.35	0.445305	Low	High
KVTKGKAKKG	0.26	0.339767	Low	High
KGKAKKGAWN	0.07	0.184903	Low	High
GGAKLKALNL	0.04	0.430832	Low	High
SKGLYRKCVK	0.09	0.145729	Low	High
KGLYRKCVKS	0.07	0.145729	Low	High
GLYRKCVKSR	0.34	0.274849	Low	High
GLLMPLKAPK	0.09	0.427961	Low	High
QRKQDDKKIK	0.15	0.861718	Low	High
RKQDDKKIKA	0.17	0.976533	Low	High
KQDDKKIKAC	0.07	0.318404	Low	High
DITFLKKDAP	0.03	0.682254	Low	Non-CPP
MLAKALRKVP	0.28	0.671110	High	High
LAKALRKVPT	0.08	0.671110	Low	High
EAKTVLKKCK	0.13	0.717688	Low	High
AKTVLKKCKS	0.13	0.717688	Low	High
KTVLKKCKSA	0.22	0.546244	Low	High
KSAFYILPSI	0.00	0.108440	Low	High
KAIVSTIQRK	0.08	0.204524	Low	High
STIQRKYKGI	0.07	0.433511	Low	High
TIQRKYKGIK	0.13	0.433511	Low	High
IQRKYKGIKI	0.08	0.433511	Low	High
GARFYFYTSK	0.04	0.178363	Low	High
ARYMRSLKVP	0.06	0.471501	Low	High
GIEFLKRGDK	0.08	0.224238	Low	High
DNLKTLLSLR	0.03	0.361469	High	Non-CPP
YMSALNHTKK	0.05	0.330689	Low	High
SALNHTKKWK	0.12	0.709364	Low	High
ALNHTKKWKY	0.10	0.888647	Low	High
LNHTKKWKYP	0.14	0.913973	Low	High
NHTKKWKYPQ	0.10	0.678789	Low	High
HTKKWKYPQV	0.04	0.303493	Low	Non-CPP
KKPASRELKV	0.14	0.769021	Low	High
KPASRELKVT	0.07	0.743805	Low	High
YTPSFKKGAK	0.08	0.164140	Low	High
PSFKKGAKLL	0.00	0.210028	Low	High
FKKGAKLLHK	0.16	0.308486	Low	High
KKGAKLLHKP	0.48	0.308486	Low	High
KGAKLLHKPI	0.32	0.261312	Low	High
WCIRCLWSTK	0.12	0.727461	High	High
CIRCLWSTKP	0.20	0.727461	Low	High
ANYAKPFLNK	0.01	0.474322	Low	High
TNIVTRCLNR	0.01	0.309069	Low	High
CTFTRSTNSR	0.02	0.513802	Low	High
TCMMCYKRNR	0.01	0.331608	High	High
MCYKRNRATR	0.25	0.936560	Low	High
CYKRNRATRV	0.03	0.936560	Low	High
YKRNRATRVE	0.00	0.936560	Low	High
KRNRATRVEC	0.18	0.532289	Low	High
RNRATRVECT	0.06	0.325111	Low	High
RDLSLQFKRP	0.17	0.903779	Low	High
SLQFKRPINP	0.14	0.353192	Low	High
HNIALIWNVK	0.01	0.377627	Low	High
LSEQLRKQIR	0.04	0.269638	Low	High
QLRKQIRSAA	0.03	0.615417	Low	High
LRKQIRSAAK	0.02	0.718721	Low	High
RKQIRSAAKK	0.30	0.751730	Low	High
KQIRSAAKKN	0.38	0.729912	Low	High
QIRSAAKKNN	0.11	0.729912	Low	High
AAKKNNLPFK	0.09	0.933084	Low	High
KKNNLPFKLT	0.04	0.938618	Low	High
NNWLKQLIKV	0.01	0.793676	High	High
LAYYFMRFRR	0.04	0.301382	Low	High
AYYFMRFRRA	0.05	0.289065	Low	High
YYFMRFRRAF	0.08	0.182381	High	High
FMRFRRAFGE	0.05	0.167797	High	High
MRFRRAFGEY	0.05	0.167797	High	High
KEMYLKLRSD	0.02	0.354324	Low	High
YNRYLALYNK	0.03	0.241742	High	High
RYLALYNKYK	0.03	0.232393	High	High
FRKMAFPSGK	0.03	0.318037	Low	High
TANPKTPKYK	0.15	0.944703	Low	High
ANPKTPKYKF	0.03	0.944703	Low	High
PKTPKYKFVR	0.12	0.944703	Low	High
KTPKYKFVRI	0.10	0.917400	Low	High
RWFLNRFTTT	0.02	0.601321	Low	High
FQSAVKRTIK	0.08	0.586710	Low	High
SEVVLKKLKK	0.24	0.204172	Low	High
VVLKKLKKSL	0.09	0.270145	Low	High
VLKKLKKSLN	0.12	0.910975	Low	High
KKLKKSLNVA	0.15	0.190690	Low	High
DAAMQRKLEK	0.00	0.372134	Low	High
AAMQRKLEKM	0.01	0.372134	Low	High
MQRKLEKMAD	0.16	0.525121	Low	High
YKQARSEDKR	0.02	0.246035	Low	High
KQARSEDKRA	0.13	0.242658	Low	High
QARSEDKRAK	0.05	0.242658	Low	High
MLFTMLRKLD	0.05	0.894360	Low	Non-CPP
QDLKWARFPK	0.02	0.860963	Low	High
DLKWARFPKS	0.08	0.860963	Low	Non-CPP
LKWARFPKSD	0.22	0.397145	Low	High
KGFCDLKGKY	0.06	0.207285	Low	High
GVSAARLTPC	0.03	0.277471	Low	High
GFAKFLKTNC	0.01	0.119116	Low	Non-CPP
KTNCCRFQEK	0.08	0.481756	Low	High
PHISRQRLTK	0.28	0.636709	Low	High
HISRQRLTKY	0.24	0.636709	Low	High
ISRQRLTKYT	0.11	0.636709	Low	High
SRQRLTKYTM	0.01	0.835148	Low	High
RQRLTKYTMA	0.11	0.878279	Low	High
GERVRQALLK	0.09	0.849631	Low	High
RVRQALLKTV	0.09	0.932330	High	High
KPYIKWDLLK	0.01	0.876722	Low	High
RLKLFDRYFK	0.05	0.261079	Low	High
KLFDRYFKYW	0.04	0.219406	High	Non-CPP
FPFNKWGKAR	0.02	0.889091	Low	High
KWGKARLYYD	0.10	0.289722	Low	High
YAISAKNRAR	0.03	0.154021	Low	High
AISAKNRART	0.05	0.241258	Low	High
KNRARTVAGV	0.12	0.254056	Low	High
NRQFHQKLLK	0.14	0.872914	Low	High
RQFHQKLLKS	0.06	0.938861	Low	High
RIMASLVLAR	0.05	0.515705	High	High
RNLQHRLYEC	0.08	0.165926	Low	Non-CPP
RLYECLYRNR	0.15	0.219346	Low	High
SLRCGACIRR	0.10	0.297578	High	High
RCGACIRRPF	0.15	0.288701	High	High
CGACIRRPFL	0.06	0.282884	High	High
GACIRRPFLC	0.19	0.252159	High	High
ACIRRPFLCC	0.11	0.249754	High	High
CIRRPFLCCK	0.31	0.306758	High	High
IRRPFLCCKC	0.11	0.355792	High	High
RRPFLCCKCC	0.18	0.274986	High	High
MSYYCKSHKP	0.01	0.306949	Low	High
ANTCTERLKL	0.02	0.144577	Low	High
SWEVGKPRPP	0.02	0.577246	Low	High
VGKPRPPLNR	0.11	0.233226	Low	High
GKPRPPLNRN	0.16	0.742499	Low	High
KALKYLPIDK	0.20	0.613457	Low	High
DKCSRIIPAR	0.01	0.588037	Low	High
KCSRIIPARA	0.05	0.577426	Low	High
CSRIIPARAR	0.07	0.565072	Low	High
SRIIPARARV	0.11	0.783032	Low	High
RIIPARARVE	0.17	0.441924	Low	High
SVVNARLRAK	0.04	0.107521	Low	High
VVNARLRAKH	0.10	0.101852	Low	High
VNARLRAKHY	0.08	0.351672	Low	High
NARLRAKHYV	0.13	0.942607	Low	High
PAPRTLLTKG	0.10	0.701705	Low	High
APRTLLTKGT	0.02	0.414738	Low	High
FNSVCRLMKT	0.01	0.084142	Low	Non-CPP
FLGTCRRCPA	0.09	0.153800	High	High
DNKLKAHKDK	0.05	0.454919	Low	High
KLKAHKDKSA	0.22	0.624144	Low	High
FLTRNPAWRK	0.17	0.889087	Low	High
RNPAWRKAVF	0.18	0.548965	Low	High
GIPKDMTYRR	0.06	0.265085	Low	High
DMTYRRLISM	0.10	0.234218	Low	Non-CPP
GNPKAIKCVP	0.05	0.253610	Low	High
WNTFTRLQSL	0.06	0.961510	Low	Non-CPP
ELWAKRNIKP	0.06	0.982818	Low	High
LWAKRNIKPV	0.01	0.982818	Low	High
WAKRNIKPVP	0.17	0.862586	Low	High
RNIKPVPEVK	0.05	0.642094	Low	High
LLIGLAKRFK	0.19	0.367884	High	High
GLAKRFKESP	0.18	0.908642	Low	High
KMQRMLLEKC	0.17	0.500000	High	High
VLRQWLPTGT	0.00	0.407571	Low	High
DMSKFPLKLR	0.11	0.167688	High	High
MSKFPLKLRG	0.02	0.319169	Low	High
SKFPLKLRGT	0.09	0.463853	Low	High
KFPLKLRGTA	0.01	0.463853	Low	High
MILSLLSKGR	0.02	0.290394	Low	High
LLSKGRLIIR	0.08	0.570179	High	High
GRLIIRENNR	0.03	0.864554	Low	High
RLIIRENNRV	0.03	0.930275	Low	High
**ORF6**				
LIIKNLSKSL	0.03	0.692987	Low	High
**ORF7a**				
HVYQLRARSV	0.00	0.333973	Low	High
QLRARSVSPK	0.18	0.788277	Low	High
RARSVSPKLF	0.06	0.540759	Low	High
RSVSPKLFIR	0.03	0.387766	Low	High
ITLCFTLKRK	0.00	0.781141	High	High
TLCFTLKRKT	0.17	0.809793	Low	High
LCFTLKRKTE	0.01	0.809793	Low	High
**ORF8**				
SKWYIRVGAR	0.06	0.354214	Low	High
KWYIRVGARK	0.15	0.337595	Low	High
YIRVGARKSA	0.13	0.276083	Low	High

* Higher scores show more possibility of cell-penetrating potential.

### Uptake efficiency analysis of the identified CPPs

The uptake efficiency of the predicted CPPs was evaluated using two different web servers such as CPPred-RF and MLCPP. These web servers classify CPPs in two categories: high or low uptake efficiency (**Table 1**).

### Calculation of peptide properties

Various physicochemical characteristics of peptides were recognized by diverse web servers such as net charge, pI, MW, amphipathicity, water solubility, hydrophobicity, hydrophobicity ratio, and polar-, non-polar-, uncharged- and charged residues. For instance, a cationic CPP can bind to cell membrane (with negative charge), then can penetrate and deliver cargoes into cells [9]. All of the physicochemical properties of CPPs were determined in **Table 2**.

**Table 2:** The properties of peptides determined by diverse web servers and tools.

Epitope	Net charge	pI	Amphipathicity	MW	Water solubility	Hydrophobicity (H)	Hydrophobic ratio by APD	Polar residues + GLY (n/%)	Nonpolar residues (n/%)	Uncharged residues + GLY	Charged residues
**S protein**											
NLTTRTQLPP	1.00	10.11	0.37	1140.31	Poor	0.379	20%	6/60.00	4/40.00	GLN 1, THR 3, ASN 1, GLY 0	ARG 1
RFQTLLALHR	2.50	12.01	0.76	1254.50	Poor	0.535	50%	5/50.00	5/50.00	GLN 1, HIS 1, THR 1, GLY 0	ARG 2
YLQPRTFLLK	2.00	10.01	0.74	1278.56	Poor	0.661	40%	4/40.00	6/60.00	GLN 1, THR 1, GLY 0	LYS 1, ARG 1
SVYAWNRKRI	3.00	11.01	0.86	1292.51	Good	0.289	40%	5/50.00	5/50.00	SER 1, ASN 1, GLY 0	LYS 1, ARG 2
YAWNRKRISN	3.00	11.01	0.86	1307.48	Good	0.107	30%	6/60.00	4/40.00	SER 1, ASN 2, GLY 0	LYS 1, ARG 2
AWNRKRISNC	3.00	10.87	0.86	1247.45	Good	0.165	40%	6/60.00	4/40.00	SER 1, ASN 2, GLY 0	LYS 1, ARG 2
WNRKRISNCV	3.00	10.87	0.86	1275.50	Good	0.256	40%	6/60.00	4/40.00	SER 1, ASN 2, GLY 0	LYS 1, ARG 2
RQIAPGQTGK	2.00	11.01	0.86	1055.20	Good	0.065	20%	7/70.00	3/30.00	GLN 2, THR 1, GLY 2	LYS 1, ARG 1
YNYLYRLFRK	3.00	10.00	0.86	1435.69	Good	0.446	30%	4/40.00	6/60.00	ASN 1, GLY 0	LYS 1, ARG 2
YLYRLFRKSN	3.00	10.29	0.86	1435.69	Good	0.346	30%	5/50.00	5/50.00	SER 1, ASN 1, GLY 0	LYS 1, ARG 2
YRLFRKSNLK	4.00	11.10	1.22	1324.59	Good	0.151	30%	6/60.00	4/40.00	SER 1, ASN 1, GLY 0	LYS 2, ARG 2
RLFRKSNLKP	4.00	12.02	1.22	1258.53	Good	0.127	30%	6/60.00	4/40.00	SER 1, ASN 1, GLY 0	LYS 2, ARG 2
RKSNLKPFER	3.00	11.01	1.35	1274.49	Good	-0.107	20%	7/70.00	3/30.00	SER 1, ASN 1, GLY 0	LYS 2, ARG 2, GLU 1
KKSTNLVKNK	4.00	10.49	1.47	1159.40	Good	-0.202	20%	8/80.00	2/20.00	SER 1, THR 1, ASN 2, GLY 0	LYS 4
KSTNLVKNKC	3.00	9.81	1.10	1134.37	Good	0.051	30%	7/70.00	3/30.00	SER 1, THR 1, ASN 2, GLY 0	LYS 3
HADQLTPTWR	0.50	7.10	0.52	1224.34	Good	0.363	30%	6/60.00	4/40.00	GLN 1, HIS 1, THR 2, GLY 0	ARG 1, ASP 1
YQTQTNSPRR	2.00	10.84	0.74	1250.34	Good	-0.090	0%	8/80.00	2/20.00	GLN 2, SER 1, THR 2, ASN 1, GLY 0	ARG 2
TQTNSPRRAR	3.00	12.31	0.86	1186.30	Good	-0.234	10%	8/80.00	2/20.00	GLN 1, SER 1, THR 2, ASN 1, GLY 0	ARG 3
TNSPRRARSV	3.00	12.31	0.74	1143.27	Good	-0.120	20%	7/70.00	3/30.00	SER 2, THR 1, ASN 1, GLY 0	ARG 3
NSPRRARSVA	3.00	12.31	0.74	1113.25	Good	-0.115	30%	6/60.00	4/40.00	SER 2, ASN 1, GLY 0	ARG 3
PRRARSVASQ	3.00	12.31	0.86	1127.27	Good	-0.077	30%	6/60.00	4/40.00	GLN 1, SER 2, GLY 0	ARG 3
KQIYKTPPIK	3.00	10.01	1.23	1215.50	Good	0.307	20%	5/50.00	5/50.00	GLN 1, THR 1, GLY 0	LYS 3
SQILPDPSKP	0.00	6.19	0.49	1081.23	Good	0.360	20%	5/50.00	5/50.00	GLN 1, SER 2, GLY 0	LYS 1, ASP 1
RLITGRLQSL	2.00	12.01	0.61	1156.39	Good	0.488	40%	6/60.00	4/40.00	GLN 1, SER 1, THR 1, GLY 1	ARG 2
**M protein**											
NRNRFLYIIK	3.00	11.01	0.86	1336.60	Good	0.384	40%	5/50.00	5/50.00	ASN 2, GLY 0	LYS 1, ARG 2
RNRFLYIIKL	3.00	11.01	0.86	1335.66	Good	0.614	50%	4/40.00	6/60.00	ASN 1, GLY 0	LYS 1, ARG 2
YIIKLIFLWL	1.00	8.94	0.37	1321.71	Poor	1.451	80%	1/10.00	9/90.00	GLY 0	LYS 1
KLIFLWLLWP	1.00	9.11	0.37	1328.71	Poor	1.462	80%	1/10.00	9/90.00	GLY 0	LYS 1
FIASFRLFAR	2.00	12.01	0.49	1227.48	Poor	0.743	70%	3/30.00	7/70.00	SER 1, GLY 0	ARG 2
ASFRLFARTR	3.00	12.31	0.74	1224.43	Good	0.309	50%	5/50.00	5/50.00	SER 1, THR 1, GLY 0	ARG 3
SFRLFARTRS	3.00	12.31	0.74	1240.43	Good	0.274	40%	6/60.00	4/40.00	SER 2, THR 1, GLY 0	ARG 3
FRLFARTRSM	3.00	12.31	0.74	1284.55	Good	0.401	50%	5/50.00	5/50.00	SER 1, THR 1, GLY 0	ARG 3
RLFARTRSMW	3.00	12.31	0.74	1323.59	Good	0.447	50%	5/50.00	5/50.00	SER 1, THR 1, GLY 0	ARG 3
FARTRSMWSF	2.00	12.01	0.49	1323.59	Poor	0.553	50%	5/50.00	5/50.00	SER 2, THR 1, GLY 0	ARG 2
HGTILTRPLL	1.50	10.11	0.39	1120.36	Poor	0.726	40%	5/50.00	5/50.00	HIS 1, THR 2, GLY 1	ARG 1
GAVILRGHLR	2.50	12.01	0.64	1091.33	Good	0.484	50%	5/50.00	5/50.00	HIS 1, GLY 2	ARG 2
RIAGHHLGRC	3.00	10.38	0.78	1119.32	Good	0.359	40%	6/60.00	4/40.00	HIS 2, GLY 2	ARG 2
YSRYRIGNYK	3.00	10.00	0.86	1319.49	Good	0.103	10%	6/60.00	4/40.00	SER 1, ASN 1, GLY 1	LYS 1, ARG 2
**N protein**											
PQNQRNAPRI	2.00	12.01	0.74	1193.33	Good	-0.011	20%	6/60.00	4/40.00	GLN 2, ASN 2, GLY 0	ARG 2
ERSGARSKQR	3.00	11.72	1.35	1174.29	Good	-0.465	10%	9/90.00	1/10.00	GLN 1, SER 2, GLY 1	LYS 1, ARG 3, GLU 1
RSGARSKQRR	5.00	12.48	1.47	1201.36	Good	-0.502	10%	9/90.00	1/10.00	GLN 1, SER 2, GLY 1	LYS 1, ARG 4
SGARSKQRRP	4.00	12.31	1.23	1142.29	Good	-0.329	10%	8/80.00	2/20.00	GLN 1, SER 2, GLY 1	LYS 1, ARG 3
GARSKQRRPQ	4.00	12.31	1.35	1183.34	Good	-0.347	10%	8/80.00	2/20.00	GLN 2, SER 1, GLY 1	LYS 1, ARG 3
ARSKQRRPQG	4.00	12.31	1.35	1183.34	Good	-0.347	10%	8/80.00	2/20.00	GLN 2, SER 1, GLY 1	LYS 1, ARG 3
RSKQRRPQGL	4.00	12.31	1.35	1225.42	Good	-0.208	10%	8/80.00	2/20.00	GLN 2, SER 1, GLY 1	LYS 1, ARG 3
SKQRRPQGLP	3.00	12.01	1.11	1166.35	Good	-0.035	10%	7/70.00	3/30.00	GLN 2, SER 1, GLY 1	LYS 1, ARG 2
KQRRPQGLPN	3.00	12.01	1.11	1193.38	Good	-0.091	10%	7/70.00	3/30.00	GLN 2, ASN 1, GLY 1	LYS 1, ARG 2
RRPQGLPNNT	2.00	12.01	0.61	1152.28	Good	-0.004	10%	7/70.00	3/30.00	GLN 1, THR 1, ASN 2, GLY 1	ARG 2
QIGYYRRATR	3.00	10.91	0.86	1283.46	Good	0.104	20%	6/60.00	4/40.00	GLN 1, THR 1, GLY 1	ARG 3
IGYYRRATRR	4.00	11.56	0.98	1311.51	Good	0.025	20%	6/60.00	4/40.00	THR 1, GLY 1	ARG 4
GYYRRATRRI	4.00	11.56	0.98	1311.51	Good	0.025	20%	6/60.00	4/40.00	THR 1, GLY 1	ARG 4
YYRRATRRIR	5.00	11.84	1.23	1410.65	Good	-0.076	20%	6/60.00	4/40.00	THR 1, GLY 0	ARG 5
YRRATRRIRG	5.00	12.18	1.23	1410.65	Good	-0.172	20%	7/70.00	3/30.00	THR 1, GLY 1	ARG 5
RRATRRIRGG	5.00	12.61	1.23	1198.40	Good	-0.268	20%	8/80.00	2/20.00	THR 1, GLY 2	ARG 5
RATRRIRGGD	3.00	12.01	0.98	1157.30	Good	-0.244	20%	8/80.00	2/20.00	THR 1, GLY 2	ARG 4, ASP 1
TRRIRGGDGK	3.00	11.72	1.10	1115.26	Good	-0.273	30%	9/90.00	1/10.00	THR 1, GLY 3	LYS 1, ARG 3, ASP 1
RIRGGDGKMK	3.00	11.01	1.22	1117.34	Good	-0.174	20%	8/80.00	2/20.00	GLY 3	LYS 2, ARG 2, ASP 1
GKMKDLSPRW	2.00	10.01	0.98	1217.46	Good	0.210	30%	6/60.00	4/40.00	SER 1, GLY 1	LYS 2, ARG 1, ASP 1
SQASSRSSSR	2.00	12.01	0.61	1052.07	Good	-0.217	10%	9/90.00	1/10.00	GLN 1, SER 6, GLY 0	ARG 2
ASSRSSSRSR	3.00	12.31	0.74	1080.13	Good	-0.296	10%	9/90.00	1/10.00	SER 6, GLY 0	ARG 3
SSRSSSRSRN	3.00	12.31	0.74	1123.15	Good	-0.387	0%	10/100.00	0/0.00	SER 6, ASN 1, GLY 0	ARG 3
SRSSSRSRNS	3.00	12.31	0.74	1123.15	Good	-0.387	0%	10/100.00	0/0.00	SER 6, ASN 1, GLY 0	ARG 3
RSSSRSRNSS	3.00	12.31	0.74	1123.15	Good	-0.387	0%	10/100.00	0/0.00	SER 6, ASN 1, GLY 0	ARG 3
SSSRSRNSSR	3.00	12.31	0.74	1123.15	Good	-0.387	0%	10/100.00	0/0.00	SER 6, ASN 1, GLY 0	ARG 3
SSRSRNSSRN	3.00	12.31	0.74	1150.18	Good	-0.443	0%	10/100.00	0/0.00	SER 5, ASN 2, GLY 0	ARG 3
RSRNSSRNST	3.00	12.31	0.74	1164.20	Good	-0.413	0%	10/100.00	0/0.00	SER 4, THR 1, ASN 2, GLY 0	ARG 3
GSSRGTSPAR	2.00	12.01	0.49	975.03	Good	-0.085	10%	8/80.00	2/20.00	SER 3, THR 1, GLY 2	ARG 2
AALALLLLDR	0.00	6.19	0.25	1068.33	Poor	0.765	80%	2/20.00	8/80.00	GLY 0	ARG 1, ASP 1
ALALLLLDRL	0.00	6.19	0.25	1110.41	Poor	0.904	80%	2/20.00	8/80.00	GLY 0	ARG 1, ASP 1
KKSAAEASKK	3.00	10.01	1.59	1047.22	Good	-0.375	30%	7/70.00	3/30.00	SER 2, GLY 0	LYS 4, GLU 1
KSAAEASKKP	2.00	9.72	1.23	1016.16	Good	-0.204	30%	6/60.00	4/40.00	SER 2, GLY 0	LYS 3, GLU 1
SAAEASKKPR	2.00	10.01	1.11	1044.18	Good	-0.206	30%	6/60.00	4/40.00	SER 2, GLY 0	LYS 2, ARG 1, GLU 1
AAEASKKPRQ	2.00	10.01	1.23	1085.23	Good	-0.224	30%	6/60.00	4/40.00	GLN 1, SER 1, GLY 0	LYS 2, ARG 1, GLU 1
AEASKKPRQK	3.00	10.30	1.60	1142.32	Good	-0.354	20%	7/70.00	3/30.00	GLN 1, SER 1, GLY 0	LYS 3, ARG 1, GLU 1
EASKKPRQKR	4.00	11.10	1.84	1227.43	Good	-0.486	10%	8/80.00	2/20.00	GLN 1, SER 1, GLY 0	LYS 3, ARG 2, GLU 1
ASKKPRQKRT	5.00	12.03	1.72	1199.42	Good	-0.396	10%	8/80.00	2/20.00	GLN 1, SER 1, THR 1, GLY 0	LYS 3, ARG 2
SKKPRQKRTA	5.00	12.03	1.72	1199.42	Good	-0.396	10%	8/80.00	2/20.00	GLN 1, SER 1, THR 1, GLY 0	LYS 3, ARG 2
KKPRQKRTAT	5.00	12.03	1.72	1213.45	Good	-0.366	10%	8/80.00	2/20.00	GLN 1, THR 2, GLY 0	LYS 3, ARG 2
KPRQKRTATK	5.00	12.03	1.72	1213.45	Good	-0.366	10%	8/80.00	2/20.00	GLN 1, THR 2, GLY 0	LYS 3, ARG 2
PRQKRTATKA	4.00	12.02	1.35	1156.35	Good	-0.236	20%	7/70.00	3/30.00	GLN 1, THR 2, GLY 0	LYS 2, ARG 2
RQKRTATKAY	4.00	11.10	1.35	1222.41	Good	-0.212	20%	7/70.00	3/30.00	GLN 1, THR 2, GLY 0	LYS 2, ARG 2
RQGTDYKHWP	1.50	8.94	0.88	1287.40	Good	0.133	10%	7/70.00	3/30.00	GLN 1, HIS 1, THR 1, GLY 1	LYS 1, ARG 1, ASP 1
FPPTEPKKDK	1.00	8.83	1.23	1186.37	Good	-0.017	10%	6/60.00	4/40.00	THR 1, GLY 0	LYS 3, GLU 1, ASP 1
PPTEPKKDKK	2.00	9.55	1.59	1167.37	Good	-0.295	0%	7/70.00	3/30.00	THR 1, GLY 0	LYS 4, GLU 1, ASP 1
PTEPKKDKKK	3.00	9.84	1.96	1198.43	Good	-0.466	0%	8/80.00	2/20.00	THR 1, GLY 0	LYS 5, GLU 1, ASP 1
TEPKKDKKKK	4.00	10.01	2.33	1229.49	Good	-0.637	0%	9/90.00	1/10.00	THR 1, GLY 0	LYS 6, GLU 1, ASP 1
EPKKDKKKKA	4.00	10.01	2.33	1199.46	Good	-0.632	10%	8/80.00	2/20.00	GLY 0	LYS 6, GLU 1, ASP 1
PKKDKKKKAD	4.00	10.01	2.20	1185.43	Good	-0.645	10%	8/80.00	2/20.00	GLY 0	LYS 6, ASP 2
KKDKKKKADE	3.00	9.71	2.33	1217.43	Good	-0.781	10%	9/90.00	1/10.00	GLY 0	LYS 6, GLU 1, ASP 2
TQALPQRQKK	3.00	11.17	1.35	1197.40	Good	-0.066	20%	7/70.00	3/30.00	GLN 3, THR 1, GLY 0	LYS 2, ARG 1
ALPQRQKKQQ	3.00	11.17	1.48	1224.43	Good	-0.114	20%	7/70.00	3/30.00	GLN 4, GLY 0	LYS 2, ARG 1
**ORF3a**											
SASKIITLKK	3.00	10.31	1.10	1088.36	Good	0.282	40%	6/60.00	4/40.00	SER 2, THR 1, GLY 0	LYS 3
ASKIITLKKR	4.00	11.27	1.35	1157.47	Good	0.185	40%	6/60.00	4/40.00	SER 1, THR 1, GLY 0	LYS 3, ARG 1
SKIITLKKRW	4.00	11.27	1.35	1272.60	Good	0.379	40%	6/60.00	4/40.00	SER 1, THR 1, GLY 0	LYS 3, ARG 1
KIITLKKRWQ	4.00	11.27	1.47	1313.65	Good	0.361	40%	6/60.00	4/40.00	GLN 1, THR 1, GLY 0	LYS 3, ARG 1
IITLKKRWQL	3.00	11.17	1.10	1298.64	Good	0.630	50%	5/50.00	5/50.00	GLN 1, THR 1, GLY 0	LYS 2, ARG 1
KKRWQLALSK	4.00	11.27	1.47	1257.55	Good	0.172	40%	6/60.00	4/40.00	GLN 1, SER 1, GLY 0	LYS 3, ARG 1
KRWQLALSKG	3.00	11.17	1.10	1186.42	Good	0.271	40%	6/60.00	4/40.00	GLN 1, SER 1, GLY 1	LYS 2, ARG 1
VRIIMRLWLC	2.00	10.38	0.49	1302.72	Poor	1.122	80%	2/20.00	8/80.00	GLY 0	ARG 2
RIIMRLWLCW	2.00	10.38	0.49	1389.80	Poor	1.225	80%	2/20.00	8/80.00	GLY 0	ARG 2
IIMRLWLCWK	2.00	9.55	0.61	1361.79	Poor	1.227	80%	2/20.00	8/80.00	GLY 0	LYS 1, ARG 1
IMRLWLCWKC	2.00	9.03	0.61	1351.77	Poor	1.201	80%	2/20.00	8/80.00	GLY 0	LYS 1, ARG 1
MRLWLCWKCR	3.00	9.72	0.86	1394.80	Good	0.920	70%	3/30.00	7/70.00	GLY 0	LYS 1, ARG 2
RLWLCWKCRS	3.00	9.72	0.86	1350.68	Good	0.793	60%	4/40.00	6/60.00	SER 1, GLY 0	LYS 1, ARG 2
LWLCWKCRSK	3.00	9.53	0.98	1322.66	Good	0.795	60%	4/40.00	6/60.00	SER 1, GLY 0	LYS 2, ARG 1
WLCWKCRSKN	3.00	9.53	0.98	1323.61	Good	0.565	50%	5/50.00	5/50.00	SER 1, ASN 1, GLY 0	LYS 2, ARG 1
LCWKCRSKNP	3.00	9.53	0.98	1234.51	Good	0.412	40%	5/50.00	5/50.00	SER 1, ASN 1, GLY 0	LYS 2, ARG 1
CWKCRSKNPL	3.00	9.53	0.98	1234.51	Good	0.412	40%	5/50.00	5/50.00	SER 1, ASN 1, GLY 0	LYS 2, ARG 1
KCRSKNPLLY	3.00	9.80	0.98	1221.49	Good	0.299	30%	5/50.00	5/50.00	SER 1, ASN 1, GLY 0	LYS 2, ARG 1
**Orf1ab**											
IKRSDARTAP	2.00	10.84	0.86	1114.27	Good	-0.042	30%	6/60.00	4/40.00	SER 1, THR 1, GLY 0	LYS 1, ARG 2, ASP 1
KRSDARTAPH	2.50	10.84	1.00	1138.25	Good	-0.209	20%	7/70.00	3/30.00	HIS 1, SER 1, THR 1, GLY 0	LYS 1, ARG 2, ASP 1
PVAYRKVLLR	3.00	11.01	0.86	1214.52	Good	0.482	50%	3/30.00	7/70.00	GLY 0	LYS 1, ARG 2
VAYRKVLLRK	4.00	11.10	1.22	1245.58	Good	0.311	50%	4/40.00	6/60.00	GLY 0	LYS 2, ARG 2
AYRKVLLRKN	4.00	11.10	1.22	1260.55	Good	0.129	40%	5/50.00	5/50.00	ASN 1, GLY 0	LYS 2, ARG 2
YRKVLLRKNG	4.00	11.10	1.22	1246.52	Good	0.098	30%	6/60.00	4/40.00	ASN 1, GLY 1	LYS 2, ARG 2
RKVLLRKNGN	4.00	12.02	1.22	1197.45	Good	-0.058	30%	7/70.00	3/30.00	ASN 2, GLY 1	LYS 2, ARG 2
KVLLRKNGNK	4.00	11.27	1.35	1169.44	Good	-0.056	30%	7/70.00	3/30.00	ASN 2, GLY 1	LYS 3, ARG 1
FEIKLAKKFD	1.00	8.83	1.23	1238.49	Good	0.301	50%	5/50.00	5/50.00	GLY 0	LYS 3, GLU 1, ASP 1
KTIQPRVEKK	3.00	10.30	1.60	1226.49	Good	-0.084	20%	7/70.00	3/30.00	GLN 1, THR 1, GLY 0	LYS 3, ARG 1, GLU 1
TIQPRVEKKK	3.00	10.30	1.60	1226.49	Good	-0.084	20%	7/70.00	3/30.00	GLN 1, THR 1, GLY 0	LYS 3, ARG 1, GLU 1
IQPRVEKKKL	3.00	10.30	1.60	1238.54	Good	0.060	30%	6/60.00	4/40.00	GLN 1, GLY 0	LYS 3, ARG 1, GLU 1
SGLKTILRKG	3.00	11.17	0.98	1072.32	Good	0.243	30%	7/70.00	3/30.00	SER 1, THR 1, GLY 2	LYS 2, ARG 1
LKTILRKGGR	4.00	12.02	1.22	1141.43	Good	0.146	30%	7/70.00	3/30.00	THR 1, GLY 2	LYS 2, ARG 2
KTILRKGGRT	4.00	12.02	1.22	1129.37	Good	0.002	20%	8/80.00	2/20.00	THR 2, GLY 2	LYS 2, ARG 2
GNFKVTKGKA	3.00	10.31	1.10	1049.24	Good	0.001	30%	7/70.00	3/30.00	THR 1, ASN 1, GLY 2	LYS 3
FKVTKGKAKK	5.00	10.61	1.84	1134.43	Good	-0.137	30%	7/70.00	3/30.00	THR 1, GLY 1	LYS 5
KVTKGKAKKG	5.00	10.61	1.84	1044.31	Good	-0.316	20%	8/80.00	2/20.00	THR 1, GLY 2	LYS 5
KGKAKKGAWN	4.00	10.49	1.47	1087.29	Good	-0.169	30%	7/70.00	3/30.00	ASN 1, GLY 2	LYS 4
GGAKLKALNL	2.00	10.02	0.73	984.21	Good	0.314	50%	5/50.00	5/50.00	ASN 1, GLY 2	LYS 2
SKGLYRKCVK	4.00	10.04	1.35	1181.47	Good	0.140	30%	6/60.00	4/40.00	SER 1, GLY 1	LYS 3, ARG 1
KGLYRKCVKS	4.00	10.04	1.35	1181.47	Good	0.140	30%	6/60.00	4/40.00	SER 1, GLY 1	LYS 3, ARG 1
GLYRKCVKSR	4.00	10.32	1.22	1209.48	Good	0.138	30%	6/60.00	4/40.00	SER 1, GLY 1	LYS 2, ARG 2
GLLMPLKAPK	2.00	10.02	0.73	1067.40	Good	0.610	50%	3/30.00	7/70.00	GLY 1	LYS 2
QRKQDDKKIK	3.00	10.01	1.96	1286.50	Good	-0.515	10%	9/90.00	1/10.00	GLN 2, GLY 0	LYS 4, ARG 1, ASP 2
RKQDDKKIKA	3.00	10.01	1.84	1229.45	Good	-0.462	20%	8/80.00	2/20.00	GLN 1, GLY 0	LYS 4, ARG 1, ASP 2
KQDDKKIKAC	2.00	9.17	1.59	1176.40	Good	-0.207	30%	7/70.00	3/30.00	GLN 1, GLY 0	LYS 4, ASP 2
DITFLKKDAP	0.00	6.31	0.73	1147.34	Good	0.306	40%	5/50.00	5/50.00	THR 1, GLY 0	LYS 2, ASP 2
MLAKALRKVP	3.00	11.17	0.98	1126.48	Good	0.420	60%	3/30.00	7/70.00	GLY 0	LYS 2, ARG 1
LAKALRKVPT	3.00	11.17	0.98	1096.38	Good	0.323	50%	4/40.00	6/60.00	THR 1, GLY 0	LYS 2, ARG 1
EAKTVLKKCK	3.00	9.65	1.59	1147.45	Good	0.043	40%	6/60.00	4/40.00	THR 1, GLY 0	LYS 4, GLU 1
AKTVLKKCKS	4.00	10.05	1.47	1105.41	Good	0.103	40%	6/60.00	4/40.00	SER 1, THR 1, GLY 0	LYS 4
KTVLKKCKSA	4.00	10.05	1.47	1105.41	Good	0.103	40%	6/60.00	4/40.00	SER 1, THR 1, GLY 0	LYS 4
KSAFYILPSI	1.00	8.94	0.37	1138.37	Poor	0.801	50%	3/30.00	7/70.00	SER 2, GLY 0	LYS 1
KAIVSTIQRK	3.00	11.17	1.10	1143.40	Good	0.214	40%	6/60.00	4/40.00	GLN 1, SER 1, THR 1, GLY 0	LYS 2, ARG 1
STIQRKYKGI	3.00	10.30	1.10	1193.41	Good	0.157	20%	7/70.00	3/30.00	GLN 1, SER 1, THR 1, GLY 1	LYS 2, ARG 1
TIQRKYKGIK	4.00	10.47	1.47	1234.5	Good	0.062	20%	7/70.00	3/30.00	GLN 1, THR 1, GLY 1	LYS 3, ARG 1
IQRKYKGIKI	4.00	10.47	1.47	1246.56	Good	0.216	30%	6/60.00	4/40.00	GLN 1, GLY 1	LYS 3, ARG 1
GARFYFYTSK	2.00	9.72	0.61	1239.40	Poor	0.403	30%	5/50.00	5/50.00	SER 1, THR 1, GLY 1	LYS 1, ARG 1
ARYMRSLKVP	3.00	11.01	0.86	1220.51	Good	0.309	40%	4/40.00	6/60.00	SER 1, GLY 0	LYS 1, ARG 2
GIEFLKRGDK	1.00	8.93	1.11	1162.35	Good	0.089	30%	7/70.00	3/30.00	GLY 2	LYS 2, ARG 1, GLU 1, ASP 1
DNLKTLLSLR	1.00	9.10	0.61	1172.39	Good	0.365	40%	6/60.00	4/40.00	SER 1, THR 1, ASN 1, GLY 0	LYS 1, ARG 1, ASP 1
YMSALNHTKK	2.50	9.72	0.88	1192.41	Good	0.197	30%	6/60.00	4/40.00	HIS 1, SER 1, THR 1, ASN 1, GLY 0	LYS 2
SALNHTKKWK	3.50	10.31	1.25	1212.42	Good	0.104	30%	7/70.00	3/30.00	HIS 1, SER 1, THR 1, ASN 1, GLY 0	LYS 3
ALNHTKKWKY	3.50	10.01	1.25	1288.52	Good	0.204	30%	6/60.00	4/40.00	HIS 1, THR 1, ASN 1, GLY 0	LYS 3
LNHTKKWKYP	3.50	10.01	1.25	1314.55	Good	0.245	20%	6/60.00	4/40.00	HIS 1, THR 1, ASN 1, GLY 0	LYS 3
NHTKKWKYPQ	3.50	10.01	1.37	1329.52	Good	0.053	10%	7/70.00	3/30.00	GLN 1, HIS 1, THR 1, ASN 1, GLY 0	LYS 3
HTKKWKYPQV	3.50	10.01	1.37	1314.55	Good	0.235	20%	6/60.00	4/40.00	GLN 1, HIS 1, THR 1, GLY 0	LYS 3
KKPASRELKV	3.00	10.30	1.47	1155.41	Good	-0.071	30%	6/60.00	4/40.00	SER 1, GLY 0	LYS 3, ARG 1, GLU 1
KPASRELKVT	2.00	10.01	1.11	1128.34	Good	0.054	30%	6/60.00	4/40.00	SER 1, THR 1, GLY 0	LYS 2, ARG 1, GLU 1
YTPSFKKGAK	3.00	10.01	1.10	1126.32	Good	0.103	20%	6/60.00	4/40.00	SER 1, THR 1, GLY 1	LYS 3
PSFKKGAKLL	3.00	10.31	1.10	1088.36	Good	0.321	40%	5/50.00	5/50.00	SER 1, GLY 1	LYS 3
FKKGAKLLHK	4.50	10.49	1.61	1169.48	Good	0.167	40%	6/60.00	4/40.00	HIS 1, GLY 1	LYS 4
KKGAKLLHKP	4.50	10.49	1.61	1119.42	Good	0.060	30%	6/60.00	4/40.00	HIS 1, GLY 1	LYS 4
KGAKLLHKPI	3.50	10.31	1.25	1104.41	Good	0.339	40%	5/50.00	5/50.00	HIS 1, GLY 1	LYS 3
WCIRCLWSTK	2.00	9.03	0.61	1295.60	Poor	0.930	60%	4/40.00	6/60.00	SER 1, THR 1, GLY 0	LYS 1, ARG 1
CIRCLWSTKP	2.00	9.03	0.61	1206.50	Poor	0.777	50%	4/40.00	6/60.00	SER 1, THR 1, GLY 0	LYS 1, ARG 1
ANYAKPFLNK	2.00	9.7	0.73	1165.36	Good	0.261	33%	4/40.00	6/60.00	ASN 2, GLY 0	LYS 2
TNIVTRCLNR	2.00	10.38	0.49	1189.40	Good	0.356	40%	6/60.00	4/40.00	THR 2, ASN 2, GLY 0	ARG 2
CTFTRSTNSR	2.00	10.38	0.49	1172.29	Good	0.141	20%	8/80.00	2/20.00	SER 2, THR 3, ASN 1, GLY 0	ARG 2
TCMMCYKRNR	3.00	9.53	0.86	1305.64	Good	0.315	40%	5/50.00	5/50.00	THR 1, ASN 1, GLY 0	LYS 1, ARG 2
MCYKRNRATR	4.00	10.92	1.10	1298.56	Good	-0.032	30%	6/60.00	4/40.00	THR 1, ASN 1, GLY 0	LYS 1, ARG 3
CYKRNRATRV	4.00	10.92	1.10	1266.49	Good	-0.033	30%	6/60.00	4/40.00	THR 1, ASN 1, GLY 0	LYS 1, ARG 3
YKRNRATRVE	3.00	10.91	1.23	1292.46	Good	-0.251	20%	7/70.00	3/30.00	THR 1, ASN 1, GLY 0	LYS 1, ARG 3, GLU 1
KRNRATRVEC	3.00	10.77	1.23	1232.43	Good	-0.193	30%	7/70.00	3/30.00	THR 1, ASN 1, GLY 0	LYS 1, ARG 3, GLU 1
RNRATRVECT	2.00	10.29	0.86	1205.36	Good	-0.068	30%	7/70.00	3/30.00	THR 2, ASN 1, GLY 0	ARG 3, GLU 1
RDLSLQFKRP	2.00	10.84	0.98	1259.47	Good	0.187	30%	6/60.00	4/40.00	GLN 1, SER 1, GLY 0	LYS 1, ARG 2, ASP 1
SLQFKRPINP	2.00	11.01	0.74	1199.42	Good	0.387	30%	5/50.00	5/50.00	GLN 1, SER 1, ASN 1, GLY 0	LYS 1, ARG 1
HNIALIWNVK	1.50	9.11	0.51	1207.44	Poor	0.702	60%	4/40.00	6/60.00	HIS 1, ASN 2, GLY 0	LYS 1
LSEQLRKQIR	2.00	10.84	1.23	1270.50	Good	0.107	30%	7/70.00	3/30.00	GLN 2, SER 1, GLY 0	LYS 1, ARG 2, GLU 1
QLRKQIRSAA	3.00	12.01	1.11	1170.38	Good	0.063	40%	6/60.00	4/40.00	GLN 2, SER 1, GLY 0	LYS 1, ARG 2
LRKQIRSAAK	4.00	12.02	1.35	1170.42	Good	-0.014	40%	6/60.00	4/40.00	GLN 1, SER 1, GLY 0	LYS 2, ARG 2
RKQIRSAAKK	5.00	12.03	1.72	1185.44	Good	-0.283	30%	7/70.00	3/30.00	GLN 1, SER 1, GLY 0	LYS 3, ARG 2
KQIRSAAKKN	4.00	11.27	1.47	1143.36	Good	-0.242	30%	7/70.00	3/30.00	GLN 1, SER 1, ASN 1, GLY 0	LYS 3, ARG 1
QIRSAAKKNN	3.00	11.17	1.10	1129.29	Good	-0.203	30%	7/70.00	3/30.00	GLN 1, SER 1, ASN 2, GLY 0	LYS 2, ARG 1
AAKKNNLPFK	3.00	10.31	1.10	1130.36	Good	0.066	40%	5/50.00	5/50.00	ASN 2, GLY 0	LYS 3
KKNNLPFKLT	3.00	10.31	1.10	1202.46	Good	0.200	30%	6/60.00	4/40.00	THR 1, ASN 2, GLY 0	LYS 3
NNWLKQLIKV	2.00	10.02	0.86	1255.53	Poor	0.527	50%	5/50.00	5/50.00	GLN 1, ASN 2, GLY 0	LYS 2
LAYYFMRFRR	3.00	10.91	0.74	1422.72	Good	0.571	50%	3/30.00	7/70.00	GLY 0	ARG 3
AYYFMRFRRA	3.00	10.91	0.74	1380.64	Good	0.432	50%	3/30.00	7/70.00	GLY 0	ARG 3
YYFMRFRRAF	3.00	10.91	0.74	1456.74	Poor	0.580	50%	3/30.00	7/70.00	GLY 0	ARG 3
FMRFRRAFGE	2.00	11.70	0.86	1316.55	Good	0.324	50%	5/50.00	5/50.00	GLY 1	ARG 3, GLU 1
MRFRRAFGEY	2.00	10.75	0.86	1332.55	Good	0.241	40%	5/50.00	5/50.00	GLY 1	ARG 3, GLU 1
KEMYLKLRSD	1.00	8.83	1.11	1282.53	Good	0.115	30%	6/60.00	4/40.00	SER 1, GLY 0	LYS 2, ARG 1, GLU 1, ASP 1
YNRYLALYNK	2.00	9.55	0.61	1317.51	Poor	0.339	30%	4/40.00	6/60.00	ASN 2, GLY 0	LYS 1, ARG 1
RYLALYNKYK	3.00	9.83	0.98	1331.58	Good	0.300	30%	4/40.00	6/60.00	ASN 1, GLY 0	LYS 2, ARG 1
FRKMAFPSGK	3.00	11.17	0.98	1168.43	Good	0.281	40%	5/50.00	5/50.00	SER 1, GLY 1	LYS 2, ARG 1
TANPKTPKYK	3.00	10.01	1.10	1147.34	Good	-0.034	10%	6/60.00	4/40.00	THR 2, ASN 1, GLY 0	LYS 3
ANPKTPKYKF	3.00	10.01	1.10	1193.41	Good	0.119	20%	5/50.00	5/50.00	THR 1, ASN 1, GLY 0	LYS 3
PKTPKYKFVR	4.00	10.47	1.35	1263.55	Good	0.169	20%	5/50.00	5/50.00	THR 1, GLY 0	LYS 3, ARG 1
KTPKYKFVRI	4.00	10.47	1.35	1279.59	Good	0.277	30%	5/50.00	5/50.00	THR 1, GLY 0	LYS 3, ARG 1
RWFLNRFTTT	2.00	12.01	0.49	1341.54	Poor	0.569	40%	6/60.00	4/40.00	THR 3, ASN 1, GLY 0	ARG 2
FQSAVKRTIK	3.00	11.17	1.10	1177.41	Good	0.213	40%	6/60.00	4/40.00	GLN 1, SER 1, THR 1, GLY 0	LYS 2, ARG 1
SEVVLKKLKK	3.00	10.01	1.59	1171.49	Good	0.120	40%	6/60.00	4/40.00	SER 1, GLY 0	LYS 4, GLU 1
VVLKKLKKSL	4.00	10.49	1.47	1155.53	Good	0.354	50%	5/50.00	5/50.00	SER 1, GLY 0	LYS 4
VLKKLKKSLN	4.00	10.49	1.47	1170.51	Good	0.172	40%	6/60.00	4/40.00	SER 1, ASN 1, GLY 0	LYS 4
KKLKKSLNVA	4.00	10.49	1.47	1128.42	Good	0.033	40%	6/60.00	4/40.00	SER 1, ASN 1, GLY 0	LYS 4
DAAMQRKLEK	1.00	8.93	1.23	1189.40	Good	-0.107	40%	6/60.00	4/40.00	GLN 1, GLY 0	LYS 2, ARG 1, GLU 1, ASP 1
AAMQRKLEKM	2.00	10.01	1.23	1205.51	Good	0.093	50%	5/50.00	5/50.00	GLN 1, GLY 0	LYS 2, ARG 1, GLU 1
MQRKLEKMAD	1.00	8.93	1.23	1249.52	Good	-0.015	40%	6/60.00	4/40.00	GLN 1, GLY 0	LYS 2, ARG 1, GLU 1, ASP 1
YKQARSEDKR	2.00	9.72	1.48	1280.41	Good	-0.440	10%	8/80.00	2/20.00	GLN 1, SER 1, GLY 0	LYS 2, ARG 2, GLU 1, ASP 1
KQARSEDKRA	2.00	10.00	1.48	1188.31	Good	-0.505	20%	8/80.00	2/20.00	GLN 1, SER 1, GLY 0	LYS 2, ARG 2, GLU 1, ASP 1
QARSEDKRAK	2.00	10.00	1.48	1188.31	Good	-0.505	20%	8/80.00	2/20.00	GLN 1, SER 1, GLY 0	LYS 2, ARG 2, GLU 1, ASP 1
MLFTMLRKLD	1.00	9.10	0.61	1267.62	Good	0.684	60%	4/40.00	6/60.00	THR 1, GLY 0	LYS 1, ARG 1, ASP 1
QDLKWARFPK	2.00	10.01	1.10	1288.52	Good	0.279	40%	5/50.00	5/50.00	GLN 1, GLY 0	LYS 2, ARG 1, ASP 1
DLKWARFPKS	2.00	10.01	0.98	1247.46	Good	0.297	40%	5/50.00	5/50.00	SER 1, GLY 0	LYS 2, ARG 1, ASP 1
LKWARFPKSD	2.00	10.01	0.98	1247.46	Good	0.297	40%	5/50.00	5/50.00	SER 1, GLY 0	LYS 2, ARG 1, ASP 1
KGFCDLKGKY	2.00	9.17	1.10	1158.39	Good	0.225	30%	6/60.00	4/40.00	GLY 2	LYS 3, ASP 1
GVSAARLTPC	1.00	8.60	0.25	974.15	Poor	0.501	50%	4/40.00	6/60.00	SER 1, THR 1, GLY 1	ARG 1
GFAKFLKTNC	2.00	9.36	0.73	1128.36	Poor	0.481	50%	5/50.00	5/50.00	THR 1, ASN 1, GLY 1	LYS 2
KTNCCRFQEK	2.00	8.98	1.23	1256.47	Good	0.068	30%	7/70.00	3/30.00	GLN 1, THR 1, ASN 1, GLY 0	LYS 2, ARG 1, GLU 1
PHISRQRLTK	3.50	12.01	1.13	1235.46	Good	0.134	20%	7/70.00	3/30.00	GLN 1, HIS 1, SER 1, THR 1, GLY 0	LYS 1, ARG 2
HISRQRLTKY	3.50	11.01	1.13	1301.52	Good	0.158	20%	7/70.00	3/30.00	GLN 1, HIS 1, SER 1, THR 1, GLY 0	LYS 1, ARG 2
ISRQRLTKYT	3.00	11.01	0.98	1265.48	Good	0.171	20%	7/70.00	3/30.00	GLN 1, SER 1, THR 2, GLY 0	LYS 1, ARG 2
SRQRLTKYTM	3.00	11.01	0.98	1283.52	Good	0.114	20%	7/70.00	3/30.00	GLN 1, SER 1, THR 2, GLY 0	LYS 1, ARG 2
RQRLTKYTMA	3.00	11.01	0.98	1267.52	Good	0.149	30%	6/60.00	4/40.00	GLN 1, THR 2, GLY 0	LYS 1, ARG 2
GERVRQALLK	2.00	10.84	1.11	1169.39	Good	0.106	40%	6/60.00	4/40.00	GLN 1, GLY 1	LYS 1, ARG 2, GLU 1
RVRQALLKTV	3.00	12.01	0.98	1183.46	Good	0.318	50%	5/50.00	5/50.00	GLN 1, THR 1, GLY 0	LYS 1, ARG 2
KPYIKWDLLK	2.00	9.55	1.10	1303.61	Good	0.539	40%	4/40.00	6/60.00	GLY 0	LYS 3, ASP 1
RLKLFDRYFK	3.00	10.29	1.22	1385.68	Good	0.317	40%	5/50.00	5/50.00	GLY 0	LYS 2, ARG 2, ASP 1
KLFDRYFKYW	2.00	9.55	0.98	1465.72	Good	0.569	40%	4/40.00	6/60.00	GLY 0	LYS 2, ARG 1, ASP 1
FPFNKWGKAR	3.00	11.17	0.98	1250.47	Good	0.327	40%	5/50.00	5/50.00	ASN 1, GLY 1	LYS 2, ARG 1
KWGKARLYYD	2.00	9.55	0.98	1299.50	Good	0.242	30%	5/50.00	5/50.00	GLY 1	LYS 2, ARG 1, ASP 1
YAISAKNRAR	3.00	11.01	0.86	1149.32	Good	0.004	40%	5/50.00	5/50.00	SER 1, ASN 1, GLY 0	LYS 1, ARG 2
AISAKNRART	3.00	12.01	0.86	1087.25	Good	-0.066	40%	6/60.00	4/40.00	SER 1, THR 1, ASN 1, GLY 0	LYS 1, ARG 2
KNRARTVAGV	3.00	12.01	0.86	1071.25	Good	-0.029	40%	6/60.00	4/40.00	THR 1, ASN 1, GLY 1	LYS 1, ARG 2
NRQFHQKLLK	3.50	11.17	1.37	1311.55	Good	0.129	30%	7/70.00	3/30.00	GLN 2, HIS 1, ASN 1, GLY 0	LYS 2, ARG 1
RQFHQKLLKS	3.50	11.17	1.37	1284.53	Good	0.185	30%	7/70.00	3/30.00	GLN 2, HIS 1, SER 1, GLY 0	LYS 2, ARG 1
RIMASLVLAR	2.00	12.01	0.49	1129.44	Poor	0.621	70%	3/30.00	7/70.00	SER 1, GLY 0	ARG 2
RNLQHRLYEC	1.50	8.57	0.89	1331.52	Good	0.255	30%	6/60.00	4/40.00	GLN 1, HIS 1, ASN 1, GLY 0	ARG 2, GLU 1
RLYECLYRNR	2.00	9.36	0.86	1385.61	Good	0.259	30%	5/50.00	5/50.00	ASN 1, GLY 0	ARG 3, GLU 1
SLRCGACIRR	3.00	10.43	0.74	1134.40	Good	0.382	50%	5/50.00	5/50.00	SER 1, GLY 1	ARG 3
RCGACIRRPF	3.00	10.43	0.74	1178.45	Good	0.467	50%	4/40.00	6/60.00	GLY 1	ARG 3
CGACIRRPFL	2.00	9.10	0.49	1135.42	Poor	0.738	60%	3/30.00	7/70.00	GLY 1	ARG 2
GACIRRPFLC	2.00	9.10	0.49	1135.42	Poor	0.738	60%	3/30.00	7/70.00	GLY 1	ARG 2
ACIRRPFLCC	2.00	8.82	0.49	1181.52	Poor	0.892	70%	2/20.00	8/80.00	GLY 0	ARG 2
CIRRPFLCCK	3.00	9.26	0.86	1238.61	Good	0.762	60%	3/30.00	7/70.00	GLY 0	LYS 1, ARG 2
IRRPFLCCKC	3.00	9.26	0.86	1238.61	Good	0.762	60%	3/30.00	7/70.00	GLY 0	LYS 1, ARG 2
RRPFLCCKCC	3.00	9.02	0.86	1228.60	Good	0.736	60%	3/30.00	7/70.00	GLY 0	LYS 1, ARG 2
MSYYCKSHKP	2.50	9.17	0.88	1243.47	Good	0.348	20%	5/50.00	5/50.00	HIS 1, SER 2, GLY 0	LYS 2
ANTCTERLKL	1.00	8.57	0.74	1148.35	Good	0.253	40%	6/60.00	4/40.00	THR 2, ASN 1, GLY 0	LYS 1, ARG 1, GLU 1
SWEVGKPRPP	1.00	9.10	0.74	1152.32	Good	0.295	20%	5/50.00	5/50.00	SER 1, GLY 1	LYS 1, ARG 1, GLU 1
VGKPRPPLNR	3.00	12.01	0.86	1133.36	Good	0.147	20%	5/50.00	5/50.00	ASN 1, GLY 1	LYS 1, ARG 2
GKPRPPLNRN	3.00	12.01	0.86	1148.33	Good	-0.035	10%	6/60.00	4/40.00	ASN 2, GLY 1	LYS 1, ARG 2
KALKYLPIDK	2.00	9.55	1.10	1188.48	Good	0.345	40%	4/40.00	6/60.00	GLY 0	LYS 3, ASP 1
DKCSRIIPAR	2.00	9.55	0.86	1158.39	Good	0.235	40%	5/50.00	5/50.00	SER 1, GLY 0	LYS 1, ARG 2, ASP 1
KCSRIIPARA	3.00	10.87	0.86	1114.38	Good	0.343	50%	4/40.00	6/60.00	SER 1, GLY 0	LYS 1, ARG 2
CSRIIPARAR	3.00	11.71	0.74	1142.39	Good	0.341	50%	4/40.00	6/60.00	SER 1, GLY 0	ARG 3
SRIIPARARV	3.00	12.31	0.74	1138.38	Good	0.309	50%	4/40.00	6/60.00	SER 1, GLY 0	ARG 3
RIIPARARVE	2.00	11.70	0.86	1180.42	Good	0.249	50%	4/40.00	6/60.00	GLY 0	ARG 3, GLU 1
SVVNARLRAK	3.00	12.01	0.86	1113.33	Good	0.111	50%	5/50.00	5/50.00	SER 1, ASN 1, GLY 0	LYS 1, ARG 2
VVNARLRAKH	3.50	12.01	1.00	1163.39	Good	0.128	50%	5/50.00	5/50.00	HIS 1, ASN 1, GLY 0	LYS 1, ARG 2
VNARLRAKHY	3.50	11.01	1.00	1227.44	Good	0.102	40%	5/50.00	5/50.00	HIS 1, ASN 1, GLY 0	LYS 1, ARG 2
NARLRAKHYV	3.50	11.01	1.00	1227.44	Good	0.102	40%	5/50.00	5/50.00	HIS 1, ASN 1, GLY 0	LYS 1, ARG 2
PAPRTLLTKG	2.00	11.01	0.61	1053.27	Good	0.367	30%	5/50.00	5/50.00	THR 2, GLY 1	LYS 1, ARG 1
APRTLLTKGT	2.00	11.01	0.61	1057.26	Good	0.321	30%	6/60.00	4/40.00	THR 3, GLY 1	LYS 1, ARG 1
FNSVCRLMKT	2.00	9.55	0.61	1198.48	Good	0.510	50%	5/50.00	5/50.00	SER 1, THR 1, ASN 1, GLY 0	LYS 1, ARG 1
FLGTCRRCPA	2.00	9.10	0.49	1123.37	Good	0.584	50%	4/40.00	6/60.00	THR 1, GLY 1	ARG 2
DNKLKAHKDK	2.50	9.55	1.61	1196.37	Good	-0.396	20%	8/80.00	2/20.00	HIS 1, ASN 1, GLY 0	LYS 4, ASP 2
KLKAHKDKSA	3.50	10.01	1.61	1125.34	Good	-0.232	30%	7/70.00	3/30.00	HIS 1, SER 1, GLY 0	LYS 4, ASP 1
FLTRNPAWRK	3.00	12.01	0.86	1288.52	Good	0.342	40%	5/50.00	5/50.00	THR 1, ASN 1, GLY 0	LYS 1, ARG 2
RNPAWRKAVF	3.00	12.01	0.86	1244.47	Good	0.299	50%	4/40.00	6/60.00	ASN 1, GLY 0	LYS 1, ARG 2
GIPKDMTYRR	2.00	10.00	0.86	1236.46	Good	0.119	20%	6/60.00	4/40.00	THR 1, GLY 1	LYS 1, ARG 2, ASP 1
DMTYRRLISM	1.00	9.10	0.49	1285.55	Good	0.435	40%	5/50.00	5/50.00	SER 1, THR 1, GLY 0	ARG 2, ASP 1
GNPKAIKCVP	2.00	9.36	0.73	1026.27	Good	0.373	40%	4/40.00	6/60.00	ASN 1, GLY 1	LYS 2
WNTFTRLQSL	1.00	10.11	0.37	1265.43	Poor	0.609	40%	6/60.00	4/40.00	GLN 1, SER 1, THR 2, ASN 1, GLY 0	ARG 1
ELWAKRNIKP	2.00	10.01	1.11	1254.50	Good	0.255	40%	5/50.00	5/50.00	ASN 1, GLY 0	LYS 2, ARG 1, GLU 1
LWAKRNIKPV	3.00	11.17	0.98	1224.52	Good	0.441	50%	4/40.00	6/60.00	ASN 1, GLY 0	LYS 2, ARG 1
WAKRNIKPVP	3.00	11.17	0.98	1208.47	Good	0.343	40%	4/40.00	6/60.00	ASN 1, GLY 0	LYS 2, ARG 1
RNIKPVPEVK	2.00	10.01	1.11	1179.43	Good	0.145	30%	5/50.00	5/50.00	ASN 1, GLY 0	LYS 2, ARG 1, GLU 1
LLIGLAKRFK	3.00	11.17	0.98	1158.50	Good	0.601	60%	4/40.00	6/60.00	GLY 1	LYS 2, ARG 1
GLAKRFKESP	2.00	10.01	1.11	1132.33	Good	0.085	30%	6/60.00	4/40.00	SER 1, GLY 1	LYS 2, ARG 1, GLU 1
KMQRMLLEKC	2.00	9.72	1.23	1279.66	Good	0.355	50%	5/50.00	5/50.00	GLN 1, GLY 0	LYS 2, ARG 1, GLU 1
VLRQWLPTGT	1.00	10.11	0.37	1170.38	Poor	0.688	40%	5/50.00	5/50.00	GLN 1, THR 2, GLY 1	ARG 1
DMSKFPLKLR	2.00	10.01	0.98	1234.53	Good	0.334	40%	5/50.00	5/50.00	SER 1, GLY 0	LYS 2, ARG 1, ASP 1
MSKFPLKLRG	3.00	11.17	0.98	1176.49	Good	0.411	40%	5/50.00	5/50.00	SER 1, GLY 1	LYS 2, ARG 1
SKFPLKLRGT	3.00	11.17	0.98	1146.40	Good	0.314	30%	6/60.00	4/40.00	SER 1, THR 1, GLY 1	LYS 2, ARG 1
KFPLKLRGTA	3.00	11.17	0.98	1130.40	Good	0.349	40%	5/50.00	5/50.00	THR 1, GLY 1	LYS 2, ARG 1
MILSLLSKGR	2.00	11.01	0.61	1117.42	Good	0.605	50%	5/50.00	5/50.00	SER 2, GLY 1	LYS 1, ARG 1
LLSKGRLIIR	3.00	12.01	0.86	1168.49	Good	0.565	50%	5/50.00	5/50.00	SER 1, GLY 1	LYS 1, ARG 2
GRLIIRENNR	2.00	11.70	0.86	1240.43	Good	0.043	30%	7/70.00	3/30.00	ASN 2, GLY 1	ARG 3, GLU 1
RLIIRENNRV	2.00	11.70	0.86	1282.51	Good	0.165	40%	6/60.00	4/40.00	ASN 2, GLY 0	ARG 3, GLU 1
**ORF6**											
LIIKNLSKSL	2.00	10.02	0.73	1128.42	Good	0.604	50%	5/50.00	5/50.00	SER 2, ASN 1, GLY 0	LYS 2
**ORF7a**											
HVYQLRARSV	2.50	10.84	0.76	1228.42	Good	0.326	40%	5/50.00	5/50.00	GLN 1, HIS 1, SER 1, GLY 0	ARG 2
QLRARSVSPK	3.00	12.01	0.98	1141.34	Good	0.064	30%	6/60.00	4/40.00	GLN 1, SER 2, GLY 0	LYS 1, ARG 2
RARSVSPKLF	3.00	12.01	0.98	1160.39	Good	0.265	40%	5/50.00	5/50.00	SER 2, GLY 0	LYS 1, ARG 2
RSVSPKLFIR	3.00	12.01	0.86	1202.47	Good	0.414	40%	5/50.00	5/50.00	SER 2, GLY 0	LYS 1, ARG 2
ITLCFTLKRK	3.00	10.07	0.98	1222.56	Good	0.606	50%	5/50.00	5/50.00	THR 2, GLY 0	LYS 2, ARG 1
TLCFTLKRKT	3.00	10.07	0.98	1210.51	Good	0.452	40%	6/60.00	4/40.00	THR 3, GLY 0	LYS 2, ARG 1
LCFTLKRKTE	2.00	9.36	1.11	1238.52	Good	0.362	40%	6/60.00	4/40.00	THR 2, GLY 0	LYS 2, ARG 1, GLU 1
**ORF8**											
SKWYIRVGAR	3.00	11.01	0.86	1235.46	Good	0.349	40%	5/50.00	5/50.00	SER 1, GLY 1	LYS 1, ARG 2
KWYIRVGARK	4.00	11.10	1.22	1276.55	Good	0.254	40%	5/50.00	5/50.00	GLY 1	LYS 2, ARG 2
YIRVGARKSA	3.00	11.01	0.86	1120.32	Good	0.155	40%	5/50.00	5/50.00	SER 1, GLY 1	LYS 1, ARG 2

### Evaluation of membrane-binding potential of CPPs

**Fig 1:**
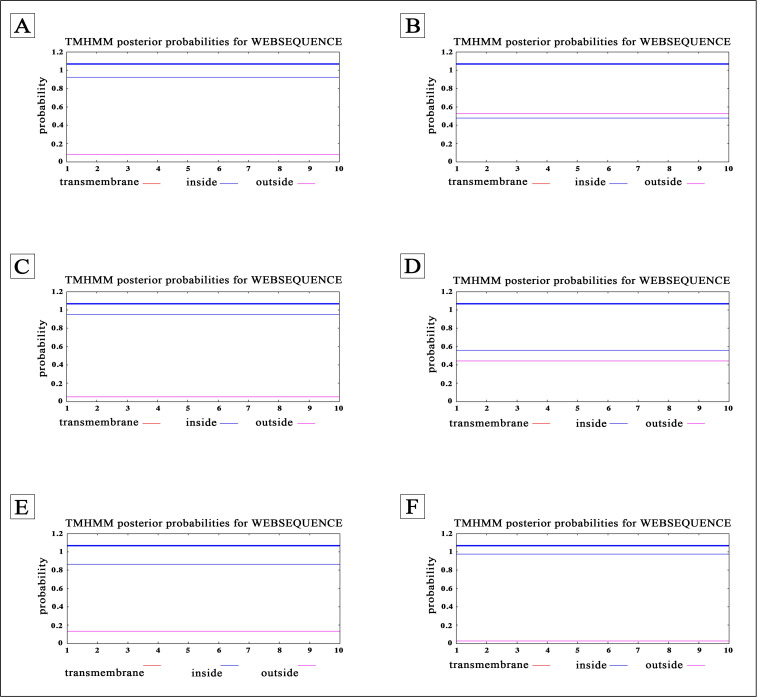
Prediction of cellular localization for CPPs using TMHMM web server: (A) EASKKPRQKR peptide containing tumor penetrating motif (RXXR); (B) GIEFLKRGDK peptide containing tumor homing motif (RGD); (C) RSGARSKQRR peptide with +5.00 net charge; (D) VVLKKLKKSL peptide with high half-life (~100 hour) in mammalian cells; (E) SSRSRNSSRN peptide with the highest Boman index (~7.5); (F) MCYKRNRATR peptide containing tumosr penetrating motif (RXXR). All of the prediction results showed the cell localization of CPPs.

One of the principal criterions to design a potent CPP is the prediction of membrane-binding ability and cellular localization. Hence, the Boman index of each peptide was estimated using APD3 web server. The values higher than 2.48 kcal/ mol define high binding potential. For example, SSRSRNSSRN peptide derived from N-protein had the highest Boman index amongst all of the predicted CPPs (Boman Index: 7.5). Moreover, the D factor was calculated for each peptide based on net charge and μH. According to the computed D factor, CPPs can be divided into three different categories including D < 0.68 as non-lipid binding (helix/random coil), 0.68 < D < 1.34 as possible lipid-binding helix, and D > 1.34 as lipid-binding helix [46]. Additionally, the cellular localization of each CPP was evaluated by TMHMM server to determine the probability of CPPs which can enter the cell. The results of membrane-binding potential and cellular localization of CPPs were indicated in **Table 3**. Also, some examples of TMHMM prediction results were illustrated in **Fig 1**.

**Table 3:** Membrane-binding potential and cellular localization of CPPs.

Epitope	Protein-binding Potential (Boman index)	Hydrophobic moment (μH)	Membrane-binding potential (D factor)	Cellular localization by TMHMM Server	Total probability of N-in by TMHMM Server
**S protein**					
NLTTRTQLPP	2.49	0.148	0.469	Inside	0.47745
RFQTLLALHR	2.3	0.342	1.147	Inside	0.48730
YLQPRTFLLK	1.09	0.225	0.8724	Outside	0.34806
SVYAWNRKRI	3.24	0.224	1.196	Inside	0.89106
YAWNRKRISN	4.31	0.162	1.142	Inside	0.91633
AWNRKRISNC	4.17	0.259	1.234	Inside	0.92814
WNRKRISNCV	3.95	0.300	1.273	Inside	0.91952
RQIAPGQTGK	2.55	0.081	0.736	Inside	0.71347
YNYLYRLFRK	2.96	0.690	1.641	Inside	0.74845
YLYRLFRKSN	3.28	0.567	1.525	Inside	0.69159
YRLFRKSNLK	3.82	0.275	1.5796	Inside	0.75816
RLFRKSNLKP	3.81	0.322	1.623	Inside	0.61242
RKSNLKPFER	4.98	0.368	1.337	Inside	0.70344
KKSTNLVKNK	3.24	0.355	1.655	Inside	0.87353
KSTNLVKNKC	2.56	0.377	1.345	Inside	0.85159
HADQLTPTWR	2.99	0.468	0.606	Inside	0.56152
YQTQTNSPRR	5.62	0.323	0.964	Inside	0.89727
TQTNSPRRAR	6.36	0.219	1.196	Inside	0.92515
TNSPRRARSV	5.49	0.292	1.265	Inside	0.85479
NSPRRARSVA	5.05	0.329	1.300	Inside	0.82719
PRRARSVASQ	4.94	0.302	1.275	Inside	0.83300
KQIYKTPPIK	1.5	0.194	1.173	Inside	0.77001
SQILPDPSKP	1.67	0.404	0.381	Outside	0.15396
RLITGRLQSL	2.07	0.502	0.897	Inside	0.47579
**M protein**					
NRNRFLYIIK	3.1	0.346	1.316	Inside	0.81684
RNRFLYIIKL	1.95	0.326	1.297	Inside	0.66588
YIIKLIFLWL	-2.91	0.488	0.790	Outside	0.20334
KLIFLWLLWP	-2.66	0.304	0.616	Outside	0.07090
FIASFRLFAR	1.08	0.522	1.152	Outside	0.32626
ASFRLFARTR	3.62	0.343	1.313	Inside	0.67733
SFRLFARTRS	4.14	0.366	1.335	Inside	0.64273
FRLFARTRSM	3.56	0.350	1.320	Inside	0.67490
RLFARTRSMW	3.63	0.589	1.546	Inside	0.78030
FARTRSMWSF	2.67	0.277	0.921	Inside	0.62650
HGTILTRPLL	0.41	0.254	0.569	Outside	0.24888
GAVILRGHLR	1.2	0.323	0.964	Outside	0.42609
RIAGHHLGRC	2.43	0.423	1.059	Inside	0.64698
YSRYRIGNYK	3.99	0.141	1.123	Inside	0.89352
**N protein**					
PQNQRNAPRI	4.74	0.243	0.889	Inside	0.80935
ERSGARSKQR	6.67	0.143	1.124	Inside	0.89938
RSGARSKQRR	7.48	0.114	1.757	Inside	0.94959
SGARSKQRRP	5.99	0.188	1.497	Inside	0.85655
GARSKQRRPQ	6.2	0.185	1.494	Inside	0.89829
ARSKQRRPQG	6.2	0.185	1.494	Inside	0.89829
RSKQRRPQGL	5.89	0.280	1.584	Inside	0.83224
SKQRRPQGLP	4.4	0.245	1.221	Inside	0.60745
KQRRPQGLPN	4.72	0.292	1.265	Inside	0.69009
RRPQGLPNNT	4.53	0.432	1.067	Inside	0.62344
QIGYYRRATR	4.54	0.383	1.351	Inside	0.93350
IGYYRRATRR	5.48	0.445	1.740	Inside	0.95251
GYYRRATRRI	5.48	0.564	1.852	Inside	0.95251
YYRRATRRIR	7.07	0.593	2.209	Inside	0.98065
YRRATRRIRG	6.96	0.565	2.183	Inside	0.96774
RRATRRIRGG	6.85	0.478	2.101	Inside	0.94656
RATRRIRGGD	6.23	0.365	1.334	Inside	0.90958
TRRIRGGDGK	5.38	0.314	1.286	Inside	0.85699
RIRGGDGKMK	3.95	0.321	1.293	Inside	0.80713
GKMKDLSPRW	2.76	0.523	1.153	Inside	0.53414
SQASSRSSSR	5.39	0.171	0.821	Inside	0.70911
ASSRSSSRSR	6.33	0.114	1.097	Inside	0.79316
SSRSSSRSRN	7.18	0.180	1.159	Inside	0.81855
SRSSSRSRNS	7.18	0.183	1.162	Inside	0.81855
RSSSRSRNSS	7.18	0.179	1.158	Inside	0.81855
SSSRSRNSSR	7.18	0.165	1.145	Inside	0.81855
SSRSRNSSRN	7.5	0.192	1.171	Inside	0.86592
RSRNSSRNST	7.42	0.194	1.173	Inside	0.90573
GSSRGTSPAR	3.89	0.156	0.807	Inside	0.52191
AALALLLLDR	-0.63	0.125	0.118	Outside	0.15140
ALALLLLDRL	-0.95	0.288	0.271	Outside	0.09169
KKSAAEASKK	3.03	0.277	1.251	Inside	0.85101
KSAAEASKKP	2.48	0.385	1.023	Inside	0.66436
SAAEASKKPR	3.42	0.284	0.928	Inside	0.71820
AAEASKKPRQ	3.63	0.306	0.948	Inside	0.79675
AEASKKPRQK	4.36	0.248	1.224	Inside	0.86032
EASKKPRQKR	6.04	0.165	1.475	Inside	0.92101
ASKKPRQKRT	5.61	0.096	1.740	Inside	0.94440
SKKPRQKRTA	5.61	0.060	1.706	Inside	0.94440
KKPRQKRTAT	5.53	0.087	1.732	Inside	0.96166
KPRQKRTATK	5.53	0.094	1.738	Inside	0.96166
PRQKRTATKA	4.79	0.202	1.510	Inside	0.94133
RQKRTATKAY	4.81	0.311	1.423	Inside	0.96930
RQGTDYKHWP	3.88	0.330	0.641	Inside	0.65834
FPPTEPKKDK	3.17	0.088	0.413	Outside	0.35377
PPTEPKKDKK	4.02	0.225	0.872	Inside	0.58321
PTEPKKDKKK	4.58	0.236	1.212	Inside	0.80442
TEPKKDKKKK	5.13	0.110	1.527	Inside	0.92302
EPKKDKKKKA	4.7	0.147	1.458	Inside	0.90887
PKKDKKKKAD	4.89	0.184	1.493	Inside	0.90457
KKDKKKKADE	5.57	0.133	1.115	Inside	0.93664
TQALPQRQKK	3.84	0.308	0.950	Inside	0.86179
ALPQRQKKQQ	4.14	0.243	1.219	Inside	0.86346
**ORF3a**					
SASKIITLKK	0.94	0.243	1.219	Inside	0.75288
ASKIITLKKR	2.09	0.314	1.452	Inside	0.86807
SKIITLKKRW	2.04	0.492	1.784	Inside	0.86095
KIITLKKRWQ	2.25	0.485	1.777	Inside	0.90139
IITLKKRWQL	1.21	0.320	1.292	Inside	0.76749
KKRWQLALSK	2.65	0.335	1.636	Inside	0.79484
KRWQLALSKG	2	0.407	1.704	Inside	0.62646
VRIIMRLWLC	0.01	0.429	1.064	Inside	0.63104
RIIMRLWLCW	0.18	0.600	1.226	Inside	0.64902
IIMRLWLCWK	-0.74	0.517	1.148	Inside	0.61977
IMRLWLCWKC	-0.38	0.347	0.987	Inside	0.68839
MRLWLCWKCR	1.59	0.156	1.137	Inside	0.81046
RLWLCWKCRS	2.17	0.178	1.158	Inside	0.77912
LWLCWKCRSK	1.23	0.223	1.200	Inside	0.74360
WLCWKCRSKN	2.39	0.080	1.065	Inside	0.85315
LCWKCRSKNP	2.62	0.198	1.176	Inside	0.78747
CWKCRSKNPL	2.62	0.236	1.212	Inside	0.78747
KCRSKNPLLY	2.5	0.134	1.116	Inside	0.67216
**Orf1ab**					
IKRSDARTAP	4.15	0.217	0.864	Inside	0.82802
KRSDARTAPH	5.11	0.039	0.696	Inside	0.81819
PVAYRKVLLR	1.58	0.313	1.285	Inside	0.60465
VAYRKVLLRK	2.13	0.208	1.516	Inside	0.80830
AYRKVLLRKN	3.2	0.224	1.531	Inside	0.85177
YRKVLLRKNG	3.29	0.241	1.547	Inside	0.79673
RKVLLRKNGN	3.94	0.200	1.508	Inside	0.80930
KVLLRKNGNK	3	0.234	1.540	Inside	0.77882
FEIKLAKKFD	1.45	0.412	0.718	Inside	0.48733
KTIQPRVEKK	3.75	0.289	1.262	Inside	0.88737
TIQPRVEKKK	3.75	0.161	1.141	Inside	0.88737
IQPRVEKKKL	3	0.103	1.087	Inside	0.78283
SGLKTILRKG	1.53	0.531	1.491	Inside	0.54410
LKTILRKGGR	2.68	0.625	1.91	Inside	0.71606
KTILRKGGRT	3.43	0.472	1.765	Inside	0.84956
GNFKVTKGKA	1.51	0.141	1.123	Inside	0.68622
FKVTKGKAKK	2.05	0.306	1.938	Inside	0.87336
KVTKGKAKKG	2.25	0.248	1.884	Inside	0.88617
KGKAKKGAWN	2.1	0.165	1.475	Inside	0.82016
GGAKLKALNL	-0.25	0.094	0.748	Outside	0.30336
SKGLYRKCVK	2.39	0.571	1.859	Inside	0.83410
KGLYRKCVKS	2.39	0.574	1.861	Inside	0.83410
GLYRKCVKSR	3.32	0.456	1.750	Inside	0.85872
GLLMPLKAPK	-0.87	0.305	0.947	Outside	0.15005
QRKQDDKKIK	6.07	0.223	1.2	Inside	0.95960
RKQDDKKIKA	5.33	0.227	1.204	Inside	0.94962
KQDDKKIKAC	3.71	0.420	1.056	Inside	0.92486
DITFLKKDAP	1.64	0.215	0.202	Inside	0.41432
MLAKALRKVP	0.61	0.641	1.595	Inside	0.57064
LAKALRKVPT	1.1	0.661	1.613	Inside	0.61542
EAKTVLKKCK	1.95	0.508	1.469	Inside	0.87393
AKTVLKKCKS	1.61	0.525	1.815	Inside	0.87056
KTVLKKCKSA	1.61	0.517	1.808	Inside	0.87056
KSAFYILPSI	-0.7	0.344	0.654	Outside	0.26827
KAIVSTIQRK	2.18	0.455	1.419	Inside	0.89354
STIQRKYKGI	2.68	0.547	1.506	Inside	0.87849
TIQRKYKGIK	2.9	0.533	1.823	Inside	0.93039
IQRKYKGIKI	2.15	0.356	1.656	Inside	0.90786
GARFYFYTSK	1.8	0.190	0.839	Inside	0.58982
ARYMRSLKVP	2.58	0.412	1.378	Inside	0.72252
GIEFLKRGDK	2.68	0.472	0.775	Inside	0.47568
DNLKTLLSLR	2.21	0.363	0.672	Outside	0.37423
YMSALNHTKK	1.94	0.373	1.012	Inside	0.74444
SALNHTKKWK	2.48	0.407	1.374	Inside	0.76830
ALNHTKKWKY	2.15	0.311	1.283	Inside	0.81149
LNHTKKWKYP	2.34	0.291	1.264	Inside	0.69869
NHTKKWKYPQ	3.38	0.362	1.331	Inside	0.82885
HTKKWKYPQV	2.31	0.217	1.194	Inside	0.78945
KKPASRELKV	3.1	0.317	1.289	Inside	0.71408
KPASRELKVT	2.8	0.356	0.996	Inside	0.66033
YTPSFKKGAK	1.7	0.425	1.391	Inside	0.60188
PSFKKGAKLL	0.44	0.488	1.450	Outside	0.28608
FKKGAKLLHK	1.12	0.639	1.923	Inside	0.56438
KKGAKLLHKP	1.42	0.544	1.833	Inside	0.55042
KGAKLLHKPI	0.37	0.590	1.546	Inside	0.43639
WCIRCLWSTK	0.93	0.223	0.870	Inside	0.79516
CIRCLWSTKP	1.17	0.298	0.941	Inside	0.70751
ANYAKPFLNK	1.64	0.408	1.045	Inside	0.53508
TNIVTRCLNR	3.3	0.562	1.190	Inside	0.89302
CTFTRSTNSR	4.67	0.140	0.792	Inside	0.87592
TCMMCYKRNR	3.74	0.228	1.205	Inside	0.96377
MCYKRNRATR	5.42	0.232	1.539	Inside	0.97415
CYKRNRATRV	5.25	0.084	1.399	Inside	0.97070
YKRNRATRVE	6.06	0.094	1.078	Inside	0.95678
KRNRATRVEC	5.92	0.077	1.062	Inside	0.96153
RNRATRVECT	5.62	0.032	0.690	Inside	0.95230
RDLSLQFKRP	4.02	0.454	1.088	Inside	0.49113
SLQFKRPINP	2.32	0.319	0.961	Outside	0.39508
HNIALIWNVK	0.05	0.188	0.507	Inside	0.60778
LSEQLRKQIR	4.19	0.686	1.307	Inside	0.75607
QLRKQIRSAA	3.64	0.527	1.487	Inside	0.86742
LRKQIRSAAK	3.64	0.621	1.906	Inside	0.89068
RKQIRSAAKK	4.68	0.451	2.075	Inside	0.95767
KQIRSAAKKN	3.86	0.413	1.709	Inside	0.93797
QIRSAAKKNN	3.97	0.319	1.291	Inside	0.92032
AAKKNNLPFK	1.84	0.198	1.176	Inside	0.63148
KKNNLPFKLT	1.96	0.238	1.214	Inside	0.54962
NNWLKQLIKV	0.87	0.728	1.347	Inside	0.65372
LAYYFMRFRR	3	0.469	1.432	Inside	0.71897
AYYFMRFRRA	3.31	0.303	1.276	Inside	0.82144
YYFMRFRRAF	3.19	0.478	1.441	Inside	0.72131
FMRFRRAFGE	3.75	0.600	1.226	Inside	0.52960
MRFRRAFGEY	4.06	0.504	1.135	Inside	0.70022
KEMYLKLRSD	3.29	0.265	0.580	Inside	0.65269
YNRYLALYNK	2.25	0.500	1.132	Inside	0.74756
RYLALYNKYK	2.14	0.392	1.360	Inside	0.80198
FRKMAFPSGK	1.83	0.313	1.285	Inside	0.47019
TANPKTPKYK	2.67	0.009	0.998	Inside	0.77069
ANPKTPKYKF	2.12	0.169	1.149	Inside	0.63687
PKTPKYKFVR	2.72	0.292	1.595	Inside	0.73005
KTPKYKFVRI	2.23	0.323	1.624	Inside	0.82704
RWFLNRFTTT	3.09	0.360	0.999	Inside	0.70898
FQSAVKRTIK	2.37	0.618	1.573	Inside	0.83635
SEVVLKKLKK	1.44	0.508	1.469	Inside	0.64932
VVLKKLKKSL	0.27	0.612	1.897	Inside	0.55852
VLKKLKKSLN	1.34	0.757	2.034	Inside	0.62971
KKLKKSLNVA	1.65	0.534	1.824	Inside	0.74490
DAAMQRKLEK	3.62	0.368	0.677	Inside	0.81844
AAMQRKLEKM	2.51	0.518	1.148	Inside	0.83287
MQRKLEKMAD	3.56	0.646	0.939	Inside	0.82292
YKQARSEDKR	6.37	0.233	0.879	Inside	0.92834
KQARSEDKRA	6.17	0.232	0.879	Inside	0.92248
QARSEDKRAK	6.17	0.186	0.835	Inside	0.92248
MLFTMLRKLD	0.93	0.562	0.860	Outside	0.39049
QDLKWARFPK	2.82	0.337	0.978	Inside	0.59730
DLKWARFPKS	2.61	0.356	0.996	Inside	0.48806
LKWARFPKSD	2.61	0.331	0.972	Inside	0.48806
KGFCDLKGKY	1.44	0.467	1.1	Inside	0.55047
GVSAARLTPC	0.6	0.109	0.432	Inside	0.44779
GFAKFLKTNC	0.53	0.530	1.160	Inside	0.51312
KTNCCRFQEK	4.2	0.333	0.974	Inside	0.92234
PHISRQRLTK	4.17	0.167	1.147	Inside	0.78638
HISRQRLTKY	4.18	0.190	1.169	Inside	0.87931
ISRQRLTKYT	3.97	0.208	1.186	Inside	0.90259
SRQRLTKYTM	4.23	0.231	1.208	Inside	0.90877
RQRLTKYTMA	3.71	0.210	1.188	Inside	0.92213
GERVRQALLK	3.11	0.401	1.038	Inside	0.66110
RVRQALLKTV	2.37	0.490	1.452	Inside	0.78888
KPYIKWDLLK	0.84	0.143	0.794	Inside	0.49602
RLKLFDRYFK	3.4	0.622	1.577	Inside	0.61889
KLFDRYFKYW	2.18	0.889	1.499	Inside	0.59390
FPFNKWGKAR	2.16	0.338	1.309	Inside	0.52642
KWGKARLYYD	2.5	0.177	0.827	Inside	0.72287
YAISAKNRAR	3.52	0.076	1.061	Inside	0.91112
AISAKNRART	3.76	0.037	1.024	Inside	0.91919
KNRARTVAGV	3.19	0.315	1.287	Inside	0.88250
NRQFHQKLLK	3.55	0.436	1.401	Inside	0.73407
RQFHQKLLKS	3.23	0.495	1.457	Inside	0.65716
RIMASLVLAR	0.84	0.180	0.829	Inside	0.57528
RNLQHRLYEC	4.25	0.519	0.819	Inside	0.77699
RLYECLYRNR	4.73	0.430	1.065	Inside	0.85428
SLRCGACIRR	3.3	0.356	1.326	Inside	0.83575
RCGACIRRPF	3.15	0.633	1.587	Inside	0.77458
CGACIRRPFL	1.17	0.422	1.058	Inside	0.52389
GACIRRPFLC	1.17	0.269	0.913	Inside	0.52389
ACIRRPFLCC	1.13	0.416	1.052	Inside	0.66539
CIRRPFLCCK	1.87	0.493	1.455	Inside	0.75457
IRRPFLCCKC	1.87	0.298	1.271	Inside	0.75457
RRPFLCCKCC	2.23	0.347	1.317	Inside	0.81002
MSYYCKSHKP	1.92	0.271	0.915	Inside	0.68047
ANTCTERLKL	2.61	0.348	0.988	Inside	0.76670
SWEVGKPRPP	2.33	0.123	0.446	Outside	0.22968
VGKPRPPLNR	3.21	0.403	1.370	Inside	0.41222
GKPRPPLNRN	4.28	0.261	1.236	Inside	0.48502
KALKYLPIDK	0.89	0.154	0.805	Inside	0.53339
DKCSRIIPAR	3.45	0.418	1.054	Inside	0.82505
KCSRIIPARA	2.4	0.376	1.344	Inside	0.83189
CSRIIPARAR	3.34	0.425	1.391	Inside	0.84740
SRIIPARARV	3.06	0.289	1.262	Inside	0.80072
RIIPARARVE	3.4	0.233	0.879	Inside	0.79867
SVVNARLRAK	2.88	0.144	1.125	Inside	0.81132
VVNARLRAKH	3.00	0.138	1.120	Inside	0.83250
VNARLRAKHY	3.42	0.100	1.084	Inside	0.86059
NARLRAKHYV	3.42	0.057	1.043	Inside	0.86059
PAPRTLLTKG	1.3	0.242	0.888	Outside	0.35023
APRTLLTKGT	1.55	0.313	0.955	Inside	0.54170
FNSVCRLMKT	1.75	0.603	1.229	Inside	0.71012
FLGTCRRCPA	1.92	0.606	1.232	Inside	0.60210
DNKLKAHKDK	4.42	0.269	0.913	Inside	0.80851
KLKAHKDKSA	3.04	0.217	1.194	Inside	0.79044
FLTRNPAWRK	3.25	0.256	1.231	Inside	0.70721
RNPAWRKAVF	2.9	0.167	1.147	Inside	0.75095
GIPKDMTYRR	3.86	0.181	0.830	Inside	0.81853
DMTYRRLISM	3.01	0.334	0.645	Inside	0.78592
GNPKAIKCVP	0.47	0.301	0.944	Inside	0.53539
WNTFTRLQSL	2.04	0.432	0.737	Inside	0.51104
ELWAKRNIKP	2.54	0.248	0.894	Inside	0.69594
LWAKRNIKPV	1.46	0.307	1.279	Inside	0.70434
WAKRNIKPVP	1.95	0.435	1.4	Inside	0.69474
RNIKPVPEVK	2.64	0.365	1.004	Inside	0.65866
LLIGLAKRFK	0.06	0.556	1.514	Outside	0.34306
GLAKRFKESP	2.55	0.567	1.195	Inside	0.41824
KMQRMLLEKC	2.25	0.534	1.164	Inside	0.77714
VLRQWLPTGT	0.84	0.442	0.747	Inside	0.37448
DMSKFPLKLR	2.29	0.438	1.073	Outside	0.39910
MSKFPLKLRG	1.33	0.425	1.391	Outside	0.33087
SKFPLKLRGT	1.82	0.312	1.284	Outside	0.37440
KFPLKLRGTA	1.3	0.337	1.308	Outside	0.41667
MILSLLSKGR	0.43	0.383	1.021	Outside	0.30296
LLSKGRLIIR	1.32	0.419	1.385	Outside	0.44682
GRLIIRENNR	4.91	0.336	0.977	Inside	0.81939
RLIIRENNRV	4.6	0.443	1.078	Inside	0.85576
**ORF6**					
LIIKNLSKSL	0	0.601	1.227	Outside	0.38147
**ORF7a**					
HVYQLRARSV	2.87	0.205	0.853	Inside	0.75260
QLRARSVSPK	3.69	0.081	1.066	Inside	0.70446
RARSVSPKLF	2.84	0.344	1.314	Inside	0.52495
RSVSPKLFIR	2.53	0.158	1.139	Inside	0.51018
ITLCFTLKRK	1.21	0.272	1.246	Inside	0.72907
TLCFTLKRKT	1.96	0.280	1.254	Inside	0.78296
LCFTLKRKTE	2.38	0.341	0.981	Inside	0.71141
**ORF8**					
SKWYIRVGAR	2.48	0.303	1.276	Inside	0.82389
KWYIRVGARK	2.7	0.208	1.516	Inside	0.89799
YIRVGARKSA	2.54	0.273	1.247	Inside	0.83725

### Assessment of the immunogenicity

As we mentioned earlier, it is important that CPPs as a delivery system should not have any immune activity. Hence, we analyzed the immunogenicity activity of each peptide using IEDB Immunogenicity Predictor. The results were listed in **Table 4**.

**Table 4:** Evaluation of immunogenicity, toxicity, allergenicity, half-life and hemolytic potency.

Epitope	Immunogenicity (IEDB)	Toxicity	Allergenicity (AllerTop)	Allergenicity (AllergenFP)	Half-life in *E*.*coli*	Half-life in mammalian	HemoPI
**S protein**							
NLTTRTQLPP	0.01498	Non-Toxin	PROBABLE ALLERGEN	PROBABLE ALLERGEN	-	-	0.49
RFQTLLALHR	0.02623	Non-Toxin	PROBABLE ALLERGEN	PROBABLE ALLERGEN	~ 2 min	~ 1 hour	0.49
YLQPRTFLLK	0.1338	Non-Toxin	PROBABLE ALLERGEN	PROBABLE ALLERGEN	~ 2 min	~ 2.8 hour	0.49
SVYAWNRKRI	0.14453	Non-Toxin	PROBABLE ALLERGEN	PROBABLE ALLERGEN	~ >10 hour	~ 1.9 hour	0.50
YAWNRKRISN	-0.02983	Non-Toxin	PROBABLE Non-ALLERGEN	PROBABLE ALLERGEN	~ 2 min	~ 2.8 hour	0.49
AWNRKRISNC	-0.12774	Non-Toxin	PROBABLE ALLERGEN	PROBABLE ALLERGEN	~ >10 min	~ 4.4 hour	0.49
WNRKRISNCV	-0.21289	Non-Toxin	PROBABLE ALLERGEN	PROBABLE Non-ALLERGEN	~ 2 min	~ 2.8 hour	0.49
RQIAPGQTGK	0.04829	Non-Toxin	PROBABLE Non-ALLERGEN	PROBABLE Non-ALLERGEN	~ 2 min	~ 1 hour	0.48
YNYLYRLFRK	0.15304	Non-Toxin	PROBABLE ALLERGEN	PROBABLE ALLERGEN	~ 2 min	~ 2.8 hour	0.49
YLYRLFRKSN	-0.07586	Non-Toxin	PROBABLE ALLERGEN	PROBABLE ALLERGEN	~ 2 min	~ 2.8 hour	0.49
YRLFRKSNLK	-0.21307	Non-Toxin	PROBABLE Non-ALLERGEN	PROBABLE Non-ALLERGEN	~ 2 min	~ 2.8 hour	0.44
RLFRKSNLKP	-0.42247	Non-Toxin	PROBABLE Non-ALLERGEN	PROBABLE Non-ALLERGEN	~ 2 min	~ 1 hour	0.49
RKSNLKPFER	-0.13415	Non-Toxin	PROBABLE Non-ALLERGEN	PROBABLE Non-ALLERGEN	~ 2 min	~ 1 hour	0.49
KKSTNLVKNK	-0.17866	Non-Toxin	PROBABLE Non-ALLERGEN	PROBABLE Non-ALLERGEN	~ 2 min	~ 1.3 hour	0.49
KSTNLVKNKC	-0.29897	Non-Toxin	PROBABLE ALLERGEN	PROBABLE Non-ALLERGEN	~ 2 min	~ 1.3 hour	0.49
HADQLTPTWR	0.06938	Non-Toxin	PROBABLE Non-ALLERGEN	PROBABLE Non-ALLERGEN	~ >10 min	~ 3.5 hour	0.49
YQTQTNSPRR	-0.20731	Non-Toxin	PROBABLE Non-ALLERGEN	PROBABLE Non-ALLERGEN	~ 2 min	~ 2.8 hour	0.49
TQTNSPRRAR	-0.05055	Non-Toxin	PROBABLE Non-ALLERGEN	PROBABLE Non-ALLERGEN	~ >10 hour	~ 7.2 hour	0.49
TNSPRRARSV	0.01979	Non-Toxin	PROBABLE ALLERGEN	PROBABLE ALLERGEN	~ >10 hour	~ 7.2 hour	0.51
NSPRRARSVA	0.0702	Non-Toxin	PROBABLE Non-ALLERGEN	PROBABLE ALLERGEN	~ >10 hour	~ 7.2 hour	0.50
PRRARSVASQ	-0.07931	Non-Toxin	PROBABLE Non-ALLERGEN	PROBABLE Non-ALLERGEN	-	-	0.49
KQIYKTPPIK	-0.07476	Non-Toxin	PROBABLE Non-ALLERGEN	PROBABLE ALLERGEN	~ >0 hour	~ >20 hour	0.49
SQILPDPSKP	-0.23322	Non-Toxin	PROBABLE ALLERGEN	PROBABLE ALLERGEN	~ >10 hour	~ 1.9 hour	0.49
RLITGRLQSL	-0.0392	Non-Toxin	PROBABLE ALLERGEN	PROBABLE ALLERGEN	~ 2 min	~ 1 hour	0.49
**M protein**							
NRNRFLYIIK	0.33978	Non-Toxin	PROBABLE ALLERGEN	PROBABLE Non-ALLERGEN	-	-	0.49
RNRFLYIIKL	0.2318	Non-Toxin	PROBABLE Non-ALLERGEN	PROBABLE Non-ALLERGEN	~ 2 min	~ 1 hour	0.50
YIIKLIFLWL	0.17526	Non-Toxin	PROBABLE Non-ALLERGEN	PROBABLE ALLERGEN	~ 2 min	~ 2.8 hour	0.49
KLIFLWLLWP	0.47552	Non-Toxin	PROBABLE ALLERGEN	PROBABLE Non-ALLERGEN	~ 2 min	~ 1.3 hour	0.49
FIASFRLFAR	0.12185	Non-Toxin	PROBABLE ALLERGEN	PROBABLE ALLERGEN	~ 2 min	~ 1.1 hour	0.49
ASFRLFARTR	0.29647	Non-Toxin	PROBABLE Non-ALLERGEN	PROBABLE Non-ALLERGEN	~ >10 min	~ 4.4 hour	0.49
SFRLFARTRS	0.26946	Non-Toxin	PROBABLE ALLERGEN	PROBABLE Non-ALLERGEN	~ >10 hour	~ 1.9 hour	0.49
FRLFARTRSM	0.18626	Non-Toxin	PROBABLE ALLERGEN	PROBABLE Non-ALLERGEN	~ 2 min	~ 1.1 hour	0.49
RLFARTRSMW	-0.02793	Non-Toxin	PROBABLE ALLERGEN	PROBABLE ALLERGEN	~ 2 min	~ 1 hour	0.49
FARTRSMWSF	-0.12986	Non-Toxin	PROBABLE ALLERGEN	PROBABLE Non-ALLERGEN	~ 2 min	~ 1.1 hour	0.49
HGTILTRPLL	0.2064	Non-Toxin	PROBABLE Non-ALLERGEN	PROBABLE ALLERGEN	~ >10 min	~ 3.5 hour	0.49
GAVILRGHLR	0.23964	Non-Toxin	PROBABLE ALLERGEN	PROBABLE ALLERGEN	~ >10 hour	~ 30 hour	0.50
RIAGHHLGRC	0.1582	Non-Toxin	PROBABLE Non-ALLERGEN	PROBABLE Non-ALLERGEN	~ 2 min	~ 1 hour	0.50
YSRYRIGNYK	0.21736	Non-Toxin	PROBABLE ALLERGEN	PROBABLE Non-ALLERGEN	~ 2 min	~ 2.8 hour	0.49
**N protein**							
PQNQRNAPRI	-0.01685	Non-Toxin	PROBABLE Non-ALLERGEN	PROBABLE Non-ALLERGEN	~ >0 hour	~ >20 hour	0.49
ERSGARSKQR	-0.33651	Non-Toxin	PROBABLE Non-ALLERGEN	PROBABLE Non-ALLERGEN	~ >10 hour	~ 1 hour	0.49
RSGARSKQRR	-0.33085	Non-Toxin	PROBABLE Non-ALLERGEN	PROBABLE Non-ALLERGEN	~ 2 min	~ 1 hour	0.49
SGARSKQRRP	-0.34144	Non-Toxin	PROBABLE Non-ALLERGEN	PROBABLE Non-ALLERGEN	~ >10 hour	~ 1.9 hour	0.49
GARSKQRRPQ	-0.38655	Non-Toxin	PROBABLE Non-ALLERGEN	PROBABLE Non-ALLERGEN	~ >10 hour	~ 30 hour	0.49
ARSKQRRPQG	-0.36142	Non-Toxin	PROBABLE Non-ALLERGEN	PROBABLE Non-ALLERGEN	~ >10 min	~ 4.4 hour	0.49
RSKQRRPQGL	-0.17416	Non-Toxin	PROBABLE Non-ALLERGEN	PROBABLE Non-ALLERGEN	~ 2 min	~ 1 hour	0.48
SKQRRPQGLP	-0.03284	Non-Toxin	PROBABLE Non-ALLERGEN	PROBABLE Non-ALLERGEN	~ >10 hour	~ 1.9 hour	0.48
KQRRPQGLPN	-0.03866	Non-Toxin	PROBABLE ALLERGEN	PROBABLE Non-ALLERGEN	~ 2 min	~ 1.3 hour	0.49
RRPQGLPNNT	-0.11764	Non-Toxin	PROBABLE ALLERGEN	PROBABLE Non-ALLERGEN	~ 2 min	~ 1 hour	0.49
QIGYYRRATR	0.1585	Non-Toxin	PROBABLE ALLERGEN	PROBABLE Non-ALLERGEN	~ >10 hour	~ 0.8 hour	0.49
IGYYRRATRR	0.19571	Non-Toxin	PROBABLE Non-ALLERGEN	PROBABLE ALLERGEN	~ >10 hour	~ 20 hour	0.49
GYYRRATRRI	0.24984	Non-Toxin	PROBABLE Non-ALLERGEN	PROBABLE Non-ALLERGEN	~ >10 hour	~ 30 hour	0.49
YYRRATRRIR	0.31494	Non-Toxin	PROBABLE Non-ALLERGEN	PROBABLE ALLERGEN	~ 2 min	~ 2.8 hour	0.49
YRRATRRIRG	0.33565	Non-Toxin	PROBABLE Non-ALLERGEN	PROBABLE Non-ALLERGEN	~ 2 min	~ 2.8 hour	0.48
RRATRRIRGG	0.34132	Non-Toxin	PROBABLE Non-ALLERGEN	PROBABLE Non-ALLERGEN	~ 2 min	~ 1 hour	0.48
RATRRIRGGD	0.3418	Non-Toxin	PROBABLE ALLERGEN	PROBABLE Non-ALLERGEN	~ 2 min	~ 1 hour	0.49
TRRIRGGDGK	0.30454	Non-Toxin	PROBABLE Non-ALLERGEN	PROBABLE ALLERGEN	~ >10 hour	~ 7.2 hour	0.49
RIRGGDGKMK	-0.1472	Non-Toxin	PROBABLE Non-ALLERGEN	PROBABLE Non-ALLERGEN	~ 2 min	~ 1 hour	0.48
GKMKDLSPRW	-0.39805	Non-Toxin	PROBABLE ALLERGEN	PROBABLE Non-ALLERGEN	~ >10 hour	~ 30 hour	0.49
SQASSRSSSR	-0.65648	Non-Toxin	PROBABLE ALLERGEN	PROBABLE Non-ALLERGEN	~ >10 hour	~ 1.9 hour	0.49
ASSRSSSRSR	-0.53253	Non-Toxin	PROBABLE ALLERGEN	PROBABLE ALLERGEN	~ >10 min	~ 4.4 hour	0.49
SSRSSSRSRN	-0.53253	Non-Toxin	PROBABLE ALLERGEN	PROBABLE Non-ALLERGEN	~ >10 hour	~ 1.9 hour	0.49
SRSSSRSRNS	-0.4467	Non-Toxin	PROBABLE ALLERGEN	PROBABLE ALLERGEN	~ >10 hour	~ 1.9 hour	0.49
RSSSRSRNSS	-0.38427	Non-Toxin	PROBABLE ALLERGEN	PROBABLE ALLERGEN	~ 2 min	~ 1 hour	0.49
SSSRSRNSSR	-0.35469	Non-Toxin	PROBABLE ALLERGEN	PROBABLE Non-ALLERGEN	~ >10 hour	~ 1.9 hour	0.49
SSRSRNSSRN	-0.37068	Non-Toxin	PROBABLE ALLERGEN	PROBABLE ALLERGEN	~ >10 hour	~ 1.9 hour	0.49
RSRNSSRNST	-0.36531	Non-Toxin	PROBABLE ALLERGEN	PROBABLE Non-ALLERGEN	~ 2 min	~ 1 hour	0.49
GSSRGTSPAR	-0.07305	Non-Toxin	PROBABLE Non-ALLERGEN	PROBABLE Non-ALLERGEN	~ >10 hour	~ 30 hour	0.49
AALALLLLDR	0.00733	Non-Toxin	PROBABLE Non-ALLERGEN	PROBABLE Non-ALLERGEN	~ >10 min	~ 4.4 hour	0.49
ALALLLLDRL	0.01846	Non-Toxin	PROBABLE Non-ALLERGEN	PROBABLE ALLERGEN	~ >10 min	~ 4.4 hour	0.49
KKSAAEASKK	-0.10752	Non-Toxin	PROBABLE ALLERGEN	PROBABLE ALLERGEN	~ 2 min	~ 1.3 hour	0.49
KSAAEASKKP	-0.27606	Non-Toxin	PROBABLE Non-ALLERGEN	PROBABLE Non-ALLERGEN	~ 2 min	~ 1.3 hour	0.49
SAAEASKKPR	-0.40103	Non-Toxin	PROBABLE Non-ALLERGEN	PROBABLE ALLERGEN	~ >10 hour	~ 1.9 hour	0.49
AAEASKKPRQ	-0.48135	Non-Toxin	PROBABLE Non-ALLERGEN	PROBABLE ALLERGEN	~ >10 min	~ 4.4 hour	0.49
AEASKKPRQK	-0.60821	Non-Toxin	PROBABLE Non-ALLERGEN	PROBABLE Non-ALLERGEN	~ >10 min	~ 4.4 hour	0.49
EASKKPRQKR	-0.66654	Non-Toxin	PROBABLE Non-ALLERGEN	PROBABLE Non-ALLERGEN	~ >10 hour	~ 1 hour	0.49
ASKKPRQKRT	-0.5082	Non-Toxin	PROBABLE Non-ALLERGEN	PROBABLE Non-ALLERGEN	~ >10 min	~ 4.4 hour	0.49
SKKPRQKRTA	-0.2802	Non-Toxin	PROBABLE Non-ALLERGEN	PROBABLE Non-ALLERGEN	~ >10 hour	~ 1.9 hour	0.49
KKPRQKRTAT	-0.16998	Non-Toxin	PROBABLE Non-ALLERGEN	PROBABLE Non-ALLERGEN	~ 2 min	~ 1.3 hour	0.49
KPRQKRTATK	-0.16712	Non-Toxin	PROBABLE Non-ALLERGEN	PROBABLE Non-ALLERGEN	~ 2 min	~ 1.3 hour	0.49
PRQKRTATKA	-0.22281	Non-Toxin	PROBABLE Non-ALLERGEN	PROBABLE ALLERGEN	~ >0 hour	~ >20 hour	0.49
RQKRTATKAY	-0.06462	Non-Toxin	PROBABLE Non-ALLERGEN	PROBABLE Non-ALLERGEN	~ 2 min	~ 1 hour	0.49
RQGTDYKHWP	0.02178	Non-Toxin	PROBABLE ALLERGEN	PROBABLE ALLERGEN	~ 2 min	~ 1 hour	0.48
FPPTEPKKDK	-0.24988	Non-Toxin	PROBABLE Non-ALLERGEN	PROBABLE ALLERGEN	~ 2 min	~ 1.1 hour	0.49
PPTEPKKDKK	-0.41773	Non-Toxin	PROBABLE Non-ALLERGEN	PROBABLE Non-ALLERGEN	~ >0 hour	~ >20 hour	0.49
PTEPKKDKKK	-0.68578	Non-Toxin	PROBABLE Non-ALLERGEN	PROBABLE ALLERGEN	~ >0 hour	~ >20 hour	0.49
TEPKKDKKKK	-0.92	Non-Toxin	PROBABLE Non-ALLERGEN	PROBABLE Non-ALLERGEN	~ >10 hour	~ 7.2 hour	0.49
EPKKDKKKKA	-0.9864	Non-Toxin	PROBABLE Non-ALLERGEN	PROBABLE Non-ALLERGEN	~ >10 hour	~ 1 hour	0.49
PKKDKKKKAD	-0.82982	Non-Toxin	PROBABLE Non-ALLERGEN	PROBABLE Non-ALLERGEN	~ >0 hour	~ >20 hour	0.49
KKDKKKKADE	-0.78682	Non-Toxin	PROBABLE Non-ALLERGEN	PROBABLE ALLERGEN	~ 2 min	~ 1.3 hour	0.49
TQALPQRQKK	-0.2971	Non-Toxin	PROBABLE Non-ALLERGEN	PROBABLE ALLERGEN	~ >10 hour	~ 7.2 hour	0.49
ALPQRQKKQQ	-0.63524	Non-Toxin	PROBABLE Non-ALLERGEN	PROBABLE ALLERGEN	~ >10 min	~ 4.4 hour	0.49
**ORF3a**							
SASKIITLKK	-0.11032	Non-Toxin	PROBABLE Non-ALLERGEN	PROBABLE Non-ALLERGEN	~ >10 hour	~ 1.9 hour	0.56
ASKIITLKKR	-0.08712	Non-Toxin	PROBABLE Non-ALLERGEN	PROBABLE ALLERGEN	~ >10 min	~ 4.4 hour	0.63
SKIITLKKRW	-0.15064	Non-Toxin	PROBABLE Non-ALLERGEN	PROBABLE ALLERGEN	~ >10 hour	~ 1.9 hour	0.49
KIITLKKRWQ	-0.16844	Non-Toxin	PROBABLE Non-ALLERGEN	PROBABLE Non-ALLERGEN	~ 2 min	~ 1.3 hour	0.48
IITLKKRWQL	-0.25058	Non-Toxin	PROBABLE Non-ALLERGEN	PROBABLE Non-ALLERGEN	~ >10 hour	~ >20 hour	0.50
KKRWQLALSK	0.0469	Non-Toxin	PROBABLE Non-ALLERGEN	PROBABLE ALLERGEN	~ 2 min	~ 1.3 hour	0.48
KRWQLALSKG	-0.29342	Non-Toxin	PROBABLE Non-ALLERGEN	PROBABLE ALLERGEN	~ 2 min	~ 1.3 hour	0.48
VRIIMRLWLC	0.22654	Non-Toxin	PROBABLE Non-ALLERGEN	PROBABLE Non-ALLERGEN	~ >10 hour	~ 100 hour	0.49
RIIMRLWLCW	0.07375	Non-Toxin	PROBABLE Non-ALLERGEN	PROBABLE Non-ALLERGEN	~ 2 min	~ 1 hour	0.49
IIMRLWLCWK	0.27346	Non-Toxin	PROBABLE ALLERGEN	PROBABLE Non-ALLERGEN	~ >10 hour	~ 20 hour	0.49
IMRLWLCWKC	0.22073	Non-Toxin	PROBABLE ALLERGEN	PROBABLE ALLERGEN	~ >10 hour	~ 20 hour	0.49
MRLWLCWKCR	0.151	Non-Toxin	PROBABLE ALLERGEN	PROBABLE Non-ALLERGEN	~ >10 hour	~ 30 hour	0.49
RLWLCWKCRS	0.00568	Toxin	PROBABLE ALLERGEN	PROBABLE Non-ALLERGEN	~ 2 min	~ 1 hour	0.49
LWLCWKCRSK	-0.15588	Toxin	PROBABLE ALLERGEN	PROBABLE Non-ALLERGEN	~ 2 min	~ 5.5 hour	0.48
WLCWKCRSKN	-0.27401	Toxin	PROBABLE Non-ALLERGEN	PROBABLE ALLERGEN	~ 2 min	~ 2.8 hour	0.49
LCWKCRSKNP	-0.48871	Toxin	PROBABLE Non-ALLERGEN	PROBABLE Non-ALLERGEN	~ 2 min	~ 5.5 hour	0.48
CWKCRSKNPL	-0.44989	Toxin	PROBABLE ALLERGEN	PROBABLE Non-ALLERGEN	~ >10 hour	~ 1.2 hour	0.48
KCRSKNPLLY	-0.39225	Toxin	PROBABLE ALLERGEN	PROBABLE Non-ALLERGEN	~ 2 min	~ 1.3 hour	0.48
**Orf1ab**							
IKRSDARTAP	0.01437	Non-Toxin	PROBABLE Non-ALLERGEN	PROBABLE Non-ALLERGEN	~ >10 hour	~ 20 hour	0.48
KRSDARTAPH	0.1202	Non-Toxin	PROBABLE Non-ALLERGEN	PROBABLE ALLERGEN	~ 2 min	~ 1.3 hour	0.49
PVAYRKVLLR	-0.1276	Non-Toxin	PROBABLE Non-ALLERGEN	PROBABLE ALLERGEN	~ >0 hour	~> 20 hour	0.49
VAYRKVLLRK	-0.10848	Non-Toxin	PROBABLE Non-ALLERGEN	PROBABLE Non-ALLERGEN	~ >0 hour	~ 100 hour	0.48
AYRKVLLRKN	-0.26356	Non-Toxin	PROBABLE Non-ALLERGEN	PROBABLE ALLERGEN	~ >10 min	~ 4.4 hour	0.48
YRKVLLRKNG	-0.18712	Non-Toxin	PROBABLE Non-ALLERGEN	PROBABLE Non-ALLERGEN	~ 2 min	~ 2.8 hour	0.48
RKVLLRKNGN	-0.14682	Non-Toxin	PROBABLE Non-ALLERGEN	PROBABLE ALLERGEN	~ 2 min	~ 1 hour	0.49
KVLLRKNGNK	-0.15563	Non-Toxin	PROBABLE Non-ALLERGEN	PROBABLE Non-ALLERGEN	~ 2 min	~ 1.3 hour	0.48
FEIKLAKKFD	-0.4631	Non-Toxin	PROBABLE ALLERGEN	PROBABLE Non-ALLERGEN	~ 2 min	~ 1.1 hour	0.47
KTIQPRVEKK	-0.0364	Non-Toxin	PROBABLE Non-ALLERGEN	PROBABLE ALLERGEN	~ 2 min	~ 1.3 hour	0.48
TIQPRVEKKK	-0.17191	Non-Toxin	PROBABLE Non-ALLERGEN	PROBABLE ALLERGEN	~ >10 min	~ 7.2 hour	0.48
IQPRVEKKKL	-0.32482	Non-Toxin	PROBABLE Non-ALLERGEN	PROBABLE ALLERGEN	~ >10 hour	~ 20 hour	0.46
SGLKTILRKG	-0.14596	Non-Toxin	PROBABLE Non-ALLERGEN	PROBABLE Non-ALLERGEN	~ >10 hour	~ 1.9 hour	0.52
LKTILRKGGR	0.03152	Non-Toxin	PROBABLE Non-ALLERGEN	PROBABLE Non-ALLERGEN	~ 2 min	~ 5.5 hour	0.47
KTILRKGGRT	-0.03682	Non-Toxin	PROBABLE Non-ALLERGEN	PROBABLE Non-ALLERGEN	~ 2 min	~ 1.3 hour	0.48
GNFKVTKGKA	-0.4014	Non-Toxin	PROBABLE ALLERGEN	PROBABLE Non-ALLERGEN	~ >10 hour	~ 30 hour	0.48
FKVTKGKAKK	-0.42052	Non-Toxin	PROBABLE Non-ALLERGEN	PROBABLE ALLERGEN	~ 2 min	~ 1.1 hour	0.49
KVTKGKAKKG	-0.65257	Non-Toxin	PROBABLE Non-ALLERGEN	PROBABLE ALLERGEN	~ 2 min	~ 1.3 hour	0.49
KGKAKKGAWN	-0.25629	Non-Toxin	PROBABLE Non-ALLERGEN	PROBABLE ALLERGEN	~ 2 min	~ 1.3 hour	0.49
GGAKLKALNL	-0.40141	Non-Toxin	PROBABLE ALLERGEN	PROBABLE ALLERGEN	~ >10 hour	~ 30 hour	0.53
SKGLYRKCVK	-0.17774	Non-Toxin	PROBABLE Non-ALLERGEN	PROBABLE ALLERGEN	~ >10 hour	~ 1.9 hour	0.47
KGLYRKCVKS	-0.30883	Non-Toxin	PROBABLE Non-ALLERGEN	PROBABLE Non-ALLERGEN	~ 2 min	~ 1.3 hour	0.47
GLYRKCVKSR	-0.45142	Non-Toxin	PROBABLE ALLERGEN	PROBABLE Non-ALLERGEN	~ >10 hour	~ 30 hour	0.45
GLLMPLKAPK	-0.37836	Non-Toxin	PROBABLE Non-ALLERGEN	PROBABLE ALLERGEN	~ >10 hour	~ 30 hour	0.49
QRKQDDKKIK	-0.4506	Non-Toxin	PROBABLE Non-ALLERGEN	PROBABLE Non-ALLERGEN	~ >10 hour	~ 0.8 hour	0.49
RKQDDKKIKA	-0.42036	Non-Toxin	PROBABLE Non-ALLERGEN	PROBABLE Non-ALLERGEN	~ 2 min	~ 1 hour	0.49
KQDDKKIKAC	-0.42434	Non-Toxin	PROBABLE ALLERGEN	PROBABLE Non-ALLERGEN	~ 2 min	~ 1.3 hour	0.49
DITFLKKDAP	-0.25182	Non-Toxin	PROBABLE ALLERGEN	PROBABLE ALLERGEN	~ >10 hour	~ 1.1 hour	0.49
MLAKALRKVP	-0.28616	Non-Toxin	PROBABLE Non-ALLERGEN	PROBABLE ALLERGEN	~ >10 hour	~ 30 hour	0.49
LAKALRKVPT	-0.16567	Non-Toxin	PROBABLE Non-ALLERGEN	PROBABLE ALLERGEN	~ 2 min	~ 5.5 hour	0.50
EAKTVLKKCK	-0.41804	Non-Toxin	PROBABLE Non-ALLERGEN	PROBABLE ALLERGEN	~ >10 hour	~ 1 hour	0.49
AKTVLKKCKS	-0.54116	Non-Toxin	PROBABLE Non-ALLERGEN	PROBABLE Non-ALLERGEN	~ >10 min	~ 4.4 hour	0.49
KTVLKKCKSA	-0.74717	Non-Toxin	PROBABLE Non-ALLERGEN	PROBABLE ALLERGEN	~ 2 min	~ 1.3 hour	0.49
KSAFYILPSI	0.14004	Non-Toxin	PROBABLE Non-ALLERGEN	PROBABLE ALLERGEN	~ 2 min	~ 1.3 hour	0.49
KAIVSTIQRK	0.0192	Non-Toxin	PROBABLE Non-ALLERGEN	PROBABLE Non-ALLERGEN	~ 2 min	~ 1.3 hour	0.49
STIQRKYKGI	-0.39864	Non-Toxin	PROBABLE Non-ALLERGEN	PROBABLE ALLERGEN	~ >10 hour	~ 1.9 hour	0.48
TIQRKYKGIK	-0.29576	Non-Toxin	PROBABLE Non-ALLERGEN	PROBABLE ALLERGEN	~ >10 hour	~ 7.2 hour	0.49
IQRKYKGIKI	-0.39558	Non-Toxin	PROBABLE Non-ALLERGEN	PROBABLE ALLERGEN	~ >10 hour	~ 20 hour	0.49
GARFYFYTSK	0.17762	Non-Toxin	PROBABLE ALLERGEN	PROBABLE Non-ALLERGEN	~ >10 hour	~ 30 hour	0.49
ARYMRSLKVP	-0.45692	Non-Toxin	PROBABLE Non-ALLERGEN	PROBABLE Non-ALLERGEN	~ >10 min	~ 4.4 hour	0.49
GIEFLKRGDK	0.01978	Non-Toxin	PROBABLE ALLERGEN	PROBABLE Non-ALLERGEN	~ >10 hour	~ 30 hour	0.47
DNLKTLLSLR	-0.35014	Non-Toxin	PROBABLE Non-ALLERGEN	PROBABLE ALLERGEN	~ >10 hour	~ 1.1 hour	0.49
YMSALNHTKK	-0.09422	Non-Toxin	PROBABLE Non-ALLERGEN	PROBABLE Non-ALLERGEN	~ 2 min	~ 2.8 hour	0.49
SALNHTKKWK	-0.19639	Non-Toxin	PROBABLE Non-ALLERGEN	PROBABLE Non-ALLERGEN	~ >10 hour	~ 1.9 hour	0.50
ALNHTKKWKY	-0.28381	Non-Toxin	PROBABLE Non-ALLERGEN	PROBABLE ALLERGEN	~ >10 min	~ 4.4 hour	0.49
LNHTKKWKYP	-0.34609	Non-Toxin	PROBABLE ALLERGEN	PROBABLE ALLERGEN	~ 2 min	~ 5.5 hour	0.49
NHTKKWKYPQ	-0.4113	Non-Toxin	PROBABLE ALLERGEN	PROBABLE ALLERGEN	-	-	0.49
HTKKWKYPQV	-0.36182	Non-Toxin	PROBABLE ALLERGEN	PROBABLE Non-ALLERGEN	~ >10 min	~ 3.5 hour	0.49
KKPASRELKV	-0.11604	Non-Toxin	PROBABLE Non-ALLERGEN	PROBABLE Non-ALLERGEN	~ 2 min	~ 1.3 hour	0.45
KPASRELKVT	-0.17419	Non-Toxin	PROBABLE Non-ALLERGEN	PROBABLE ALLERGEN	~ 2 min	~ 1.3 hour	0.49
YTPSFKKGAK	-0.41761	Non-Toxin	PROBABLE Non-ALLERGEN	PROBABLE ALLERGEN	~ 2 min	~ 2.8 hour	0.49
PSFKKGAKLL	-0.50765	Non-Toxin	PROBABLE Non-ALLERGEN	PROBABLE Non-ALLERGEN	~ >0 hour	~ >20 hour	0.51
FKKGAKLLHK	-0.2087	Non-Toxin	PROBABLE Non-ALLERGEN	PROBABLE ALLERGEN	~ 2 min	~ 1.1 hour	0.48
KKGAKLLHKP	-0.27957	Non-Toxin	PROBABLE Non-ALLERGEN	PROBABLE Non-ALLERGEN	~ 2 min	~ 1.3 hour	0.47
KGAKLLHKPI	-0.38393	Non-Toxin	PROBABLE Non-ALLERGEN	PROBABLE Non-ALLERGEN	~ 2 min	~ 1.3 hour	0.44
WCIRCLWSTK	0.12355	Toxin	PROBABLE Non-ALLERGEN	PROBABLE ALLERGEN	~ 2 min	~ 2.8 hour	0.49
CIRCLWSTKP	-0.08152	Non-Toxin	PROBABLE Non-ALLERGEN	PROBABLE Non-ALLERGEN	~ >10 hour	~ 1.2 hour	0.48
ANYAKPFLNK	-0.08557	Non-Toxin	PROBABLE Non-ALLERGEN	PROBABLE ALLERGEN	~ >10 min	~ 4.4 hour	0.49
TNIVTRCLNR	0.10905	Non-Toxin	PROBABLE ALLERGEN	PROBABLE ALLERGEN	~ >10 hour	~ 7.2 hour	0.49
CTFTRSTNSR	-0.09922	Non-Toxin	PROBABLE Non-ALLERGEN	PROBABLE ALLERGEN	~ >10 hour	~ 1.2 hour	0.49
TCMMCYKRNR	-0.4529	Toxin	PROBABLE Non-ALLERGEN	PROBABLE Non-ALLERGEN	~ >10 hour	~ 7.2 hour	0.49
MCYKRNRATR	-0.06968	Toxin	PROBABLE ALLERGEN	PROBABLE ALLERGEN	~ >10 hour	~ 30 hour	0.48
CYKRNRATRV	0.12601	Toxin	PROBABLE Non-ALLERGEN	PROBABLE ALLERGEN	~ >10 hour	~ 1.2 hour	0.49
YKRNRATRVE	0.20313	Non-Toxin	PROBABLE Non-ALLERGEN	PROBABLE Non-ALLERGEN	~ 2 min	~ 2.8 hour	0.49
KRNRATRVEC	0.26794	Non-Toxin	PROBABLE Non-ALLERGEN	PROBABLE ALLERGEN	~ 2 min	~ 1.3 hour	0.49
RNRATRVECT	0.23623	Non-Toxin	PROBABLE Non-ALLERGEN	PROBABLE ALLERGEN	~ 2 min	~ 1 hour	0.49
RDLSLQFKRP	-0.33523	Non-Toxin	PROBABLE Non-ALLERGEN	PROBABLE Non-ALLERGEN	~ 2 min	~ 1 hour	0.49
SLQFKRPINP	0.0187	Non-Toxin	PROBABLE ALLERGEN	PROBABLE Non-ALLERGEN	~ >10 hour	~ 1.9 hour	0.48
HNIALIWNVK	0.42854	Non-Toxin	PROBABLE Non-ALLERGEN	PROBABLE Non-ALLERGEN	~ >10 min	~ 3.5 hour	0.49
LSEQLRKQIR	-0.26746	Non-Toxin	PROBABLE Non-ALLERGEN	PROBABLE Non-ALLERGEN	~ 2 min	~ 5.5 hour	0.49
QLRKQIRSAA	-0.25144	Non-Toxin	PROBABLE ALLERGEN	PROBABLE ALLERGEN	~ >10 min	~ 0.8 hour	0.44
LRKQIRSAAK	-0.10641	Non-Toxin	PROBABLE ALLERGEN	PROBABLE ALLERGEN	~ 2 min	~ 5.5 hour	0.37
RKQIRSAAKK	-0.07053	Non-Toxin	PROBABLE Non-ALLERGEN	PROBABLE Non-ALLERGEN	~ 2 min	~ 1 hour	0.48
KQIRSAAKKN	-0.29889	Non-Toxin	PROBABLE Non-ALLERGEN	PROBABLE ALLERGEN	~ 2 min	~ 1.3 hour	0.50
QIRSAAKKNN	-0.46225	Non-Toxin	PROBABLE Non-ALLERGEN	PROBABLE ALLERGEN	~ >10 hour	~ 0.8 hour	0.49
AAKKNNLPFK	-0.251	Non-Toxin	PROBABLE Non-ALLERGEN	PROBABLE ALLERGEN	~ >10 min	~ 4.4 hour	0.49
KKNNLPFKLT	-0.10849	Non-Toxin	PROBABLE ALLERGEN	PROBABLE Non-ALLERGEN	~ 2 min	~ 1.3 hour	0.49
NNWLKQLIKV	-0.28618	Non-Toxin	PROBABLE Non-ALLERGEN	PROBABLE Non-ALLERGEN	-	-	0.49
LAYYFMRFRR	0.11584	Non-Toxin	PROBABLE Non-ALLERGEN	PROBABLE ALLERGEN	~ 2 min	~ 5.5 hour	0.49
AYYFMRFRRA	0.18012	Non-Toxin	PROBABLE Non-ALLERGEN	PROBABLE ALLERGEN	~ >10 min	~ 4.4 hour	0.49
YYFMRFRRAF	0.14096	Non-Toxin	PROBABLE ALLERGEN	PROBABLE ALLERGEN	~ 2 min	~ 2.8 hour	0.49
FMRFRRAFGE	0.39083	Non-Toxin	PROBABLE ALLERGEN	PROBABLE Non-ALLERGEN	~ 2 min	~ 1.1 hour	0.49
MRFRRAFGEY	0.37588	Non-Toxin	PROBABLE ALLERGEN	PROBABLE Non-ALLERGEN	~ >10 hour	~ 30 hour	0.49
KEMYLKLRSD	-0.34494	Non-Toxin	PROBABLE Non-ALLERGEN	PROBABLE ALLERGEN	~ 2 min	~ 1.3 hour	0.49
YNRYLALYNK	0.02304	Non-Toxin	PROBABLE ALLERGEN	PROBABLE ALLERGEN	~ 2 min	~ 2.8 hour	0.49
RYLALYNKYK	-0.16888	Non-Toxin	PROBABLE ALLERGEN	PROBABLE ALLERGEN	~ 2 min	~ 1 hour	0.49
FRKMAFPSGK	-0.22486	Non-Toxin	PROBABLE ALLERGEN	PROBABLE Non-ALLERGEN	~ 2 min	~ 1.1 hour	0.50
TANPKTPKYK	-0.38006	Non-Toxin	PROBABLE Non-ALLERGEN	PROBABLE ALLERGEN	~ >10 hour	~ 7.2 hour	0.49
ANPKTPKYKF	-0.52572	Non-Toxin	PROBABLE Non-ALLERGEN	PROBABLE ALLERGEN	~ >10 min	~ 4.4 hour	0.49
PKTPKYKFVR	-0.29224	Non-Toxin	PROBABLE Non-ALLERGEN	PROBABLE Non-ALLERGEN	~ >0 hour	~ >30 hour	0.49
KTPKYKFVRI	-0.25892	Non-Toxin	PROBABLE Non-ALLERGEN	PROBABLE Non-ALLERGEN	~ 2 min	~ 1.3 hour	0.49
RWFLNRFTTT	0.23658	Non-Toxin	PROBABLE ALLERGEN	PROBABLE ALLERGEN	~ 2 min	~ 1 hour	0.49
FQSAVKRTIK	-0.02489	Non-Toxin	PROBABLE ALLERGEN	PROBABLE ALLERGEN	~ 2 min	~ 1.1 hour	0.50
SEVVLKKLKK	-0.50422	Non-Toxin	PROBABLE ALLERGEN	PROBABLE ALLERGEN	~ >10 hour	~ 1.9 hour	0.48
VVLKKLKKSL	-0.92306	Non-Toxin	PROBABLE Non-ALLERGEN	PROBABLE Non-ALLERGEN	~ >10 hour	~ 100 hour	0.48
VLKKLKKSLN	-0.8569	Non-Toxin	PROBABLE Non-ALLERGEN	PROBABLE ALLERGEN	~ >10 hour	~ 100 hour	0.48
KKLKKSLNVA	-0.58348	Non-Toxin	PROBABLE Non-ALLERGEN	PROBABLE ALLERGEN	~ 2 min	~ 1.3 hour	0.47
DAAMQRKLEK	-0.38026	Non-Toxin	PROBABLE Non-ALLERGEN	PROBABLE Non-ALLERGEN	~ >10 hour	~ 1.1 hour	0.49
AAMQRKLEKM	-0.3851	Non-Toxin	PROBABLE Non-ALLERGEN	PROBABLE Non-ALLERGEN	~ >10 min	~ 4.4 hour	0.49
MQRKLEKMAD	-0.44184	Non-Toxin	PROBABLE ALLERGEN	PROBABLE ALLERGEN	~ >10 hour	~ 30 hour	0.49
YKQARSEDKR	-0.12196	Non-Toxin	PROBABLE Non-ALLERGEN	PROBABLE Non-ALLERGEN	~ 2 min	~ 2.8 hour	0.49
KQARSEDKRA	-0.1297	Non-Toxin	PROBABLE Non-ALLERGEN	PROBABLE Non-ALLERGEN	~ 2 min	~ 1.3 hour	0.49
QARSEDKRAK	-0.16703	Non-Toxin	PROBABLE Non-ALLERGEN	PROBABLE Non-ALLERGEN	~ >10 hour	~ 0.8 hour	0.49
MLFTMLRKLD	-0.2445	Non-Toxin	PROBABLE Non-ALLERGEN	PROBABLE Non-ALLERGEN	~ >10 hour	~ 30 hour	0.49
QDLKWARFPK	0.17424	Non-Toxin	PROBABLE Non-ALLERGEN	PROBABLE Non-ALLERGEN	~ >10 hour	~ 0.8 hour	0.48
DLKWARFPKS	0.21623	Non-Toxin	PROBABLE ALLERGEN	PROBABLE Non-ALLERGEN	~ >10 hour	~ 1.1 hour	0.49
LKWARFPKSD	-0.01343	Non-Toxin	PROBABLE ALLERGEN	PROBABLE Non-ALLERGEN	~ 2 min	~ 5.5 hour	0.49
KGFCDLKGKY	-0.30585	Non-Toxin	PROBABLE Non-ALLERGEN	PROBABLE ALLERGEN	~ 2 min	~ 1.3 hour	0.49
GVSAARLTPC	0.09001	Non-Toxin	PROBABLE ALLERGEN	PROBABLE Non-ALLERGEN	~ >10 hour	~ 30 hour	0.48
GFAKFLKTNC	-0.27512	Non-Toxin	PROBABLE ALLERGEN	PROBABLE Non-ALLERGEN	~ >10 hour	~ 30 hour	0.49
KTNCCRFQEK	0.01249	Toxin	PROBABLE Non-ALLERGEN	PROBABLE ALLERGEN	~ 2 min	~ 1.3 hour	0.48
PHISRQRLTK	-0.12363	Non-Toxin	PROBABLE ALLERGEN	PROBABLE Non-ALLERGEN	~ >0 hour	~ >20 hour	0.47
HISRQRLTKY	-0.1677	Non-Toxin	PROBABLE Non-ALLERGEN	PROBABLE Non-ALLERGEN	~ >10 min	~ 3.5 hour	0.48
ISRQRLTKYT	-0.20778	Non-Toxin	PROBABLE Non-ALLERGEN	PROBABLE ALLERGEN	~ >10 hour	~ 20 hour	0.49
SRQRLTKYTM	-0.14196	Non-Toxin	PROBABLE Non-ALLERGEN	PROBABLE ALLERGEN	~ >10 hour	~ 1.9 hour	0.49
RQRLTKYTMA	-0.23988	Non-Toxin	PROBABLE Non-ALLERGEN	PROBABLE ALLERGEN	~ 2 min	~ 1 hour	0.49
GERVRQALLK	0.01693	Non-Toxin	PROBABLE Non-ALLERGEN	PROBABLE ALLERGEN	~ >10 hour	~ 30 hour	0.48
RVRQALLKTV	-0.24222	Non-Toxin	PROBABLE Non-ALLERGEN	PROBABLE ALLERGEN	~ 2 min	~ 1 hour	0.48
KPYIKWDLLK	0.14346	Non-Toxin	PROBABLE ALLERGEN	PROBABLE Non-ALLERGEN	~ 2 min	~ 1.3 hour	0.48
RLKLFDRYFK	0.16844	Non-Toxin	PROBABLE ALLERGEN	PROBABLE ALLERGEN	~ 2 min	~ 1 hour	0.46
KLFDRYFKYW	0.03316	Non-Toxin	PROBABLE Non-ALLERGEN	PROBABLE ALLERGEN	~ 2 min	~ 1.3 hour	0.49
FPFNKWGKAR	-0.09005	Non-Toxin	PROBABLE ALLERGEN	PROBABLE ALLERGEN	~ 2 min	~ 1.1 hour	0.49
KWGKARLYYD	-0.13322	Non-Toxin	PROBABLE Non-ALLERGEN	PROBABLE ALLERGEN	~ 2 min	~ 1.3 hour	0.49
YAISAKNRAR	-0.23472	Non-Toxin	PROBABLE ALLERGEN	PROBABLE Non-ALLERGEN	~ 2 min	~ 2.8 hour	0.49
AISAKNRART	-0.11865	Non-Toxin	PROBABLE Non-ALLERGEN	PROBABLE ALLERGEN	~ >10 min	~ 4.4 hour	0.49
KNRARTVAGV	0.23605	Non-Toxin	PROBABLE Non-ALLERGEN	PROBABLE Non-ALLERGEN	~ 2 min	~ 1.3 hour	0.49
NRQFHQKLLK	-0.21994	Non-Toxin	PROBABLE Non-ALLERGEN	PROBABLE Non-ALLERGEN	-	-	0.49
RQFHQKLLKS	-0.39805	Non-Toxin	PROBABLE Non-ALLERGEN	PROBABLE Non-ALLERGEN	~ 2 min	~ 1 hour	0.49
RIMASLVLAR	-0.13717	Non-Toxin	PROBABLE ALLERGEN	PROBABLE Non-ALLERGEN	~ 2 min	~ 1 hour	0.48
RNLQHRLYEC	0.00668	Toxin	PROBABLE Non-ALLERGEN	PROBABLE Non-ALLERGEN	~ 2 min	~ 1 hour	0.49
RLYECLYRNR	0.07267	Toxin	PROBABLE Non-ALLERGEN	PROBABLE ALLERGEN	~ 2 min	~ 1 hour	0.49
SLRCGACIRR	0.12546	Non-Toxin	PROBABLE ALLERGEN	PROBABLE Non-ALLERGEN	~ >10 hour	~ 1.9 hour	0.46
RCGACIRRPF	0.21339	Non-Toxin	PROBABLE ALLERGEN	PROBABLE Non-ALLERGEN	~ 2 min	~ 1 hour	0.48
CGACIRRPFL	0.24621	Non-Toxin	PROBABLE ALLERGEN	PROBABLE Non-ALLERGEN	~ >10 hour	~ 1.2 hour	0.43
GACIRRPFLC	0.2991	Non-Toxin	PROBABLE Non-ALLERGEN	PROBABLE Non-ALLERGEN	~ >10 hour	~ 30 hour	0.43
ACIRRPFLCC	0.20422	Toxin	PROBABLE Non-ALLERGEN	PROBABLE ALLERGEN	~ >10 min	~ 4.4 hour	0.48
CIRRPFLCCK	0.08464	Toxin	PROBABLE Non-ALLERGEN	PROBABLE Non-ALLERGEN	~ >10 hour	~ 1.2 hour	0.49
IRRPFLCCKC	-0.11341	Toxin	PROBABLE ALLERGEN	PROBABLE Non-ALLERGEN	~ >10 hour	~ 20 hour	0.49
RRPFLCCKCC	-0.21335	Toxin	PROBABLE Non-ALLERGEN	PROBABLE ALLERGEN	~ 2 min	~ 1 hour	0.49
MSYYCKSHKP	-0.52185	Non-Toxin	PROBABLE ALLERGEN	PROBABLE Non-ALLERGEN	~ >10 hour	~ 30 hour	0.49
ANTCTERLKL	0.00701	Non-Toxin	PROBABLE Non-ALLERGEN	PROBABLE Non-ALLERGEN	~ >10 min	~ 4.4 hour	0.49
SWEVGKPRPP	-0.0762	Non-Toxin	PROBABLE ALLERGEN	PROBABLE Non-ALLERGEN	~ >10 hour	~ 1.9 hour	0.49
VGKPRPPLNR	-0.06514	Non-Toxin	PROBABLE Non-ALLERGEN	PROBABLE ALLERGEN	~ >10 hour	~ 100 hour	0.49
GKPRPPLNRN	0.04122	Non-Toxin	PROBABLE Non-ALLERGEN	PROBABLE ALLERGEN	~ >10 hour	~ 30 hour	0.49
KALKYLPIDK	-0.12016	Non-Toxin	PROBABLE ALLERGEN	PROBABLE ALLERGEN	~ 2 min	~ 1.3 hour	0.48
DKCSRIIPAR	0.13481	Non-Toxin	PROBABLE Non-ALLERGEN	PROBABLE ALLERGEN	~ >10 hour	~ 1.1 hour	0.48
KCSRIIPARA	0.3104	Non-Toxin	PROBABLE Non-ALLERGEN	PROBABLE Non-ALLERGEN	~ 2 min	~ 1.3 hour	0.48
CSRIIPARAR	0.37289	Non-Toxin	PROBABLE ALLERGEN	PROBABLE ALLERGEN	~ >10 hour	~ 1.2 hour	0.49
SRIIPARARV	0.3164	Non-Toxin	PROBABLE Non-ALLERGEN	PROBABLE ALLERGEN	~ >10 hour	~ 1.9 hour	0.49
RIIPARARVE	0.22517	Non-Toxin	PROBABLE Non-ALLERGEN	PROBABLE ALLERGEN	~ 2 min	~ 1 hour	0.49
SVVNARLRAK	0.15149	Non-Toxin	PROBABLE Non-ALLERGEN	PROBABLE ALLERGEN	~ >10 hour	~ 1.9 hour	0.48
VVNARLRAKH	0.03261	Non-Toxin	PROBABLE Non-ALLERGEN	PROBABLE ALLERGEN	~ >10 hour	~ 100 hour	0.49
VNARLRAKHY	-0.02189	Non-Toxin	PROBABLE ALLERGEN	PROBABLE ALLERGEN	~ >10 hour	~ 100 hour	0.48
NARLRAKHYV	-0.08372	Non-Toxin	PROBABLE Non-ALLERGEN	PROBABLE ALLERGEN	-	-	0.48
PAPRTLLTKG	-0.0282	Non-Toxin	PROBABLE Non-ALLERGEN	PROBABLE ALLERGEN	~ >0 hour	~ >20 hour	0.49
APRTLLTKGT	-0.0914	Non-Toxin	PROBABLE Non-ALLERGEN	PROBABLE Non-ALLERGEN	~ >10 min	~ 4.4 hour	0.49
FNSVCRLMKT	-0.2989	Non-Toxin	PROBABLE ALLERGEN	PROBABLE ALLERGEN	~ 2 min	~ 1.1 hour	0.48
FLGTCRRCPA	0.0447	Non-Toxin	PROBABLE ALLERGEN	PROBABLE Non-ALLERGEN	~ 2 min	~ 1.1 hour	0.47
DNKLKAHKDK	-0.39165	Non-Toxin	PROBABLE Non-ALLERGEN	PROBABLE ALLERGEN	~ >10 hour	~ 1.1 hour	0.49
KLKAHKDKSA	-0.46691	Non-Toxin	PROBABLE Non-ALLERGEN	PROBABLE Non-ALLERGEN	~ 2 min	~ 1.3 hour	0.48
FLTRNPAWRK	0.30159	Non-Toxin	PROBABLE Non-ALLERGEN	PROBABLE Non-ALLERGEN	~ 2 min	~ 1.1 hour	0.48
RNPAWRKAVF	0.15601	Non-Toxin	PROBABLE Non-ALLERGEN	PROBABLE ALLERGEN	~ 2 min	~ 1 hour	0.48
GIPKDMTYRR	-0.30634	Non-Toxin	PROBABLE ALLERGEN	PROBABLE Non-ALLERGEN	~ >10 hour	~ 30 hour	0.49
DMTYRRLISM	0.1149	Non-Toxin	PROBABLE Non-ALLERGEN	PROBABLE ALLERGEN	~ >10 hour	~ 1.1 hour	0.49
GNPKAIKCVP	-0.27728	Non-Toxin	PROBABLE Non-ALLERGEN	PROBABLE Non-ALLERGEN	~ >10 hour	~ 30 hour	0.48
WNTFTRLQSL	0.01374	Non-Toxin	PROBABLE Non-ALLERGEN	PROBABLE Non-ALLERGEN	~ 2 min	~ 2.8 hour	0.49
ELWAKRNIKP	-0.0681	Non-Toxin	PROBABLE ALLERGEN	PROBABLE Non-ALLERGEN	~ >10 hour	~ 1 hour	0.49
LWAKRNIKPV	-0.2234	Non-Toxin	PROBABLE ALLERGEN	PROBABLE Non-ALLERGEN	~ 2 min	~ 5.5 hour	0.50
WAKRNIKPVP	-0.08286	Non-Toxin	PROBABLE ALLERGEN	PROBABLE ALLERGEN	~ 2 min	~ 2.8 hour	0.49
RNIKPVPEVK	-0.04622	Non-Toxin	PROBABLE ALLERGEN	PROBABLE ALLERGEN	~ 2 min	~ 1 hour	0.48
LLIGLAKRFK	0.01368	Non-Toxin	PROBABLE ALLERGEN	PROBABLE ALLERGEN	~ 2 min	~ 5.5 hour	0.52
GLAKRFKESP	-0.25506	Non-Toxin	PROBABLE Non-ALLERGEN	PROBABLE ALLERGEN	~ >10 hour	~ 30 hour	0.52
KMQRMLLEKC	-0.21926	Non-Toxin	PROBABLE Non-ALLERGEN	PROBABLE Non-ALLERGEN	~ 2 min	~ 1.3 hour	0.49
VLRQWLPTGT	0.14726	Non-Toxin	PROBABLE ALLERGEN	PROBABLE Non-ALLERGEN	~ >10 hour	~ 100 hour	0.49
DMSKFPLKLR	-0.36642	Non-Toxin	PROBABLE Non-ALLERGEN	PROBABLE ALLERGEN	~ >10 hour	~ 1.1 hour	0.49
MSKFPLKLRG	-0.15592	Non-Toxin	PROBABLE ALLERGEN	PROBABLE ALLERGEN	~ >10 hour	~ 30 hour	0.52
SKFPLKLRGT	-0.14092	Non-Toxin	PROBABLE ALLERGEN	PROBABLE Non-ALLERGEN	~ >10 hour	~ 1.9 hour	0.53
KFPLKLRGTA	-0.13556	Non-Toxin	PROBABLE Non-ALLERGEN	PROBABLE Non-ALLERGEN	~ 2 min	~ 1.3 hour	0.52
MILSLLSKGR	-0.5096	Non-Toxin	PROBABLE Non-ALLERGEN	PROBABLE Non-ALLERGEN	~ >10 hour	~ 30 hour	0.50
LLSKGRLIIR	-0.00766	Non-Toxin	PROBABLE ALLERGEN	PROBABLE Non-ALLERGEN	~ 2 min	~ 5.5 hour	0.50
GRLIIRENNR	0.39533	Non-Toxin	PROBABLE Non-ALLERGEN	PROBABLE Non-ALLERGEN	~ >10 hour	~ 30 hour	0.49
RLIIRENNRV	0.34371	Non-Toxin	PROBABLE Non-ALLERGEN	PROBABLE ALLERGEN	~ 2 min	~ 1 hour	0.49
**ORF6**							
LIIKNLSKSL	-0.62529	Non-Toxin	PROBABLE ALLERGEN	PROBABLE Non-ALLERGEN	~ 2 min	~ 5.5 hour	0.49
**ORF7a**							
HVYQLRARSV	-0.09431	Non-Toxin	PROBABLE Non-ALLERGEN	PROBABLE Non-ALLERGEN	~ >10 min	~ 3.5 hour	0.49
QLRARSVSPK	-0.16177	Non-Toxin	PROBABLE ALLERGEN	PROBABLE ALLERGEN	~ >10 hour	~ 0.8 hour	0.47
RARSVSPKLF	-0.46949	Non-Toxin	PROBABLE Non-ALLERGEN	PROBABLE Non-ALLERGEN	~ 2 min	~ 1 hour	0.48
RSVSPKLFIR	-0.20775	Non-Toxin	PROBABLE Non-ALLERGEN	PROBABLE Non-ALLERGEN	~ 2 min	~ 1 hour	0.48
ITLCFTLKRK	-0.06825	Non-Toxin	PROBABLE Non-ALLERGEN	PROBABLE Non-ALLERGEN	~ >10 hour	~ 20 hour	0.49
TLCFTLKRKT	-0.15802	Non-Toxin	PROBABLE Non-ALLERGEN	PROBABLE Non-ALLERGEN	~ >10 hour	~ 7.2 hour	0.49
LCFTLKRKTE	-0.25434	Non-Toxin	PROBABLE Non-ALLERGEN	PROBABLE Non-ALLERGEN	~ 2 min	~ 5.5 hour	0.49
**ORF8**							
SKWYIRVGAR	0.3385	Non-Toxin	PROBABLE Non-ALLERGEN	PROBABLE Non-ALLERGEN	~ >10 hour	~ 1.9 hour	0.49
KWYIRVGARK	0.31848	Non-Toxin	PROBABLE Non-ALLERGEN	PROBABLE ALLERGEN	~ 2 min	~ 1.3 hour	0.49
YIRVGARKSA	-0.1005	Non-Toxin	PROBABLE Non-ALLERGEN	PROBABLE Non-ALLERGEN	~ 2 min	~ 2.8 hour	0.48

### Determination of toxicity and allergenicity

The toxicity and allergenicity of each peptide were determined using diverse web servers (**Table 4**). In detail, most of the predicted CPPs were non-toxic. The toxic CPPs were derived from ORF3a and Orf1ab polyproteins. Furthermore, there are some differences between allergenicity prediction by AllerTop, and AllergenFP web servers. Some CPPs were determined as probable allergen by AllerTop, whereas they were identified as probable non-allergen by AllergenFP. It is rational to select CPPs which were determined as probable non-allergen by both web servers.

### Estimation of hemolytic potency and half-life

It should be considered that high hydrophobicity of a peptide enhances its probability of hydrolysis in the host; therefore, the probability of hydrolysis and half-life of each peptide in *E*.*coli* and mammalian were evaluated using HemoPI and ProtLifePred web servers (**Table 4**). The results of hemolytic potency vary between 0 and 1 (*i*.*e*., 0 very unlikely to be hemolytic, and 1 very likely to be hemolytic). For example, seven predicted CPPs had the highest half-life in mammalian cells (~ 100 hours) which all of them were derived from Orf1ab polyprotein including VAYRKVLLRK, VVLKKLKKSL, VLKKLKKSLN, VGKPRPPLNR, VVNARLRAKH, VNARLRAKHY, and VLRQWLPTGT peptides.

### Prediction of CPP structure

**Fig 2:**
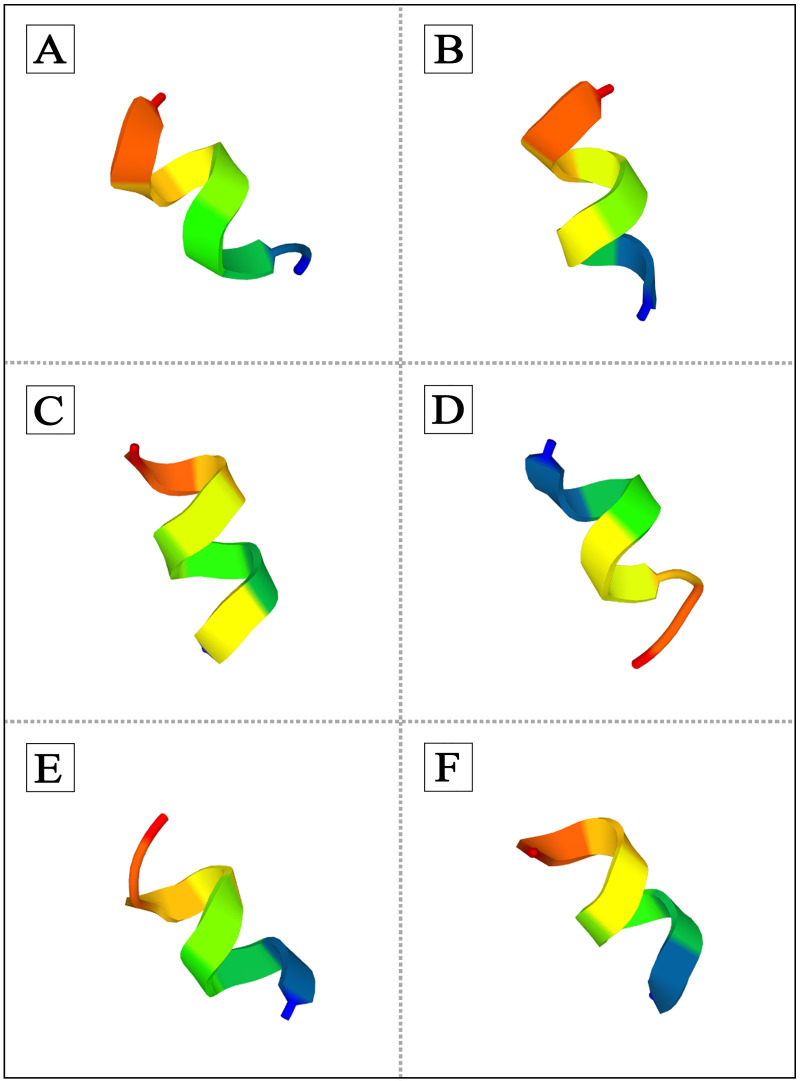
The 3D spatial shape of CPPs predicted by PEP-FOLD3 web server: (A) EASKKPRQKR peptide containing tumor penetrating motif (RXXR); (B) GIEFLKRGDK peptide containing tumor homing motif (RGD); (C) RSGARSKQRR peptide with +5.00 net charge; (D) VVLKKLKKSL peptide with high half-life (~100 hour) in mammalian cells; (E) SSRSRNSSRN peptide with the highest Boman index (~7.5); (F) MCYKRNRATR peptide containing tumor penetrating motif (RXXR).

**Fig 3:**
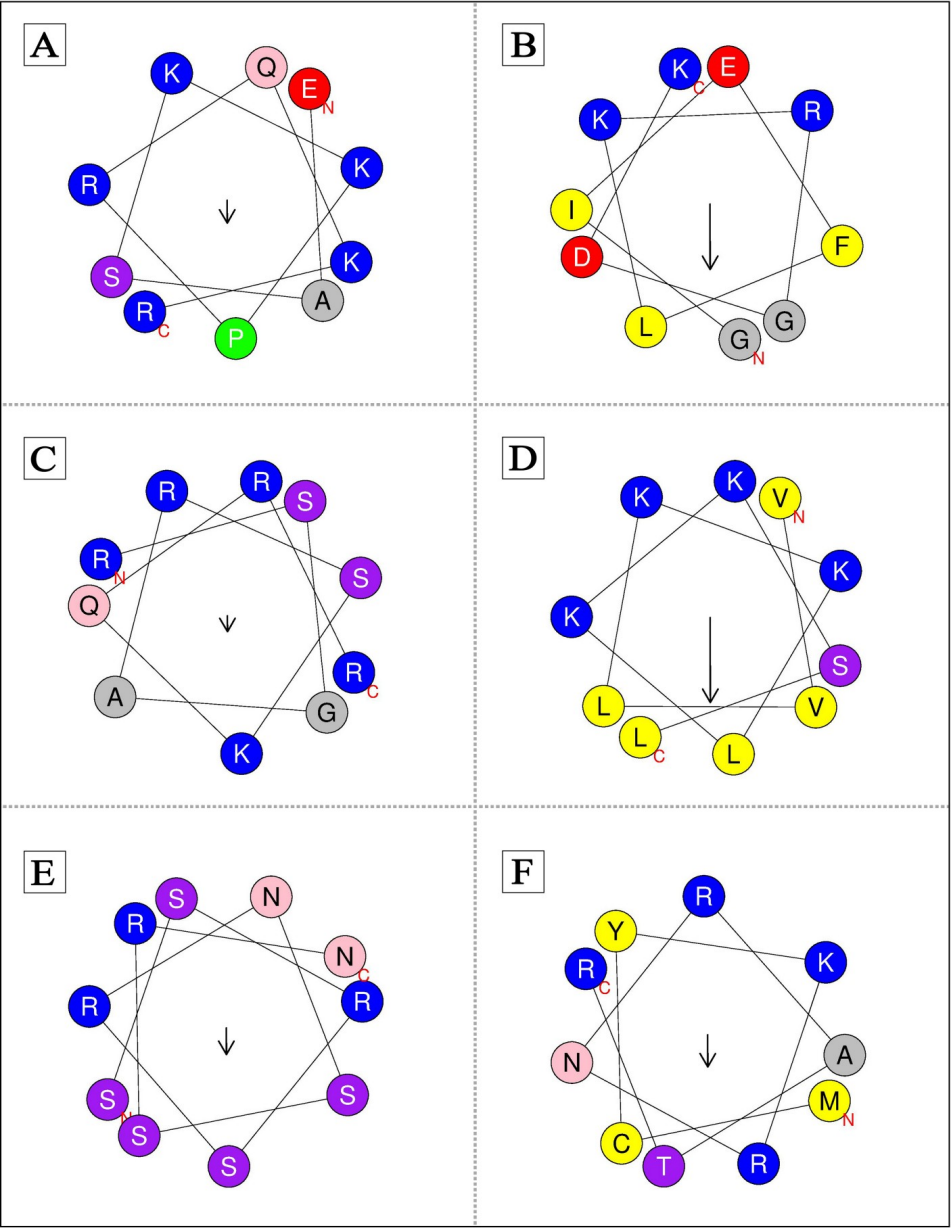
Helical wheel projection of the six selected CPPs by HeliQuest: These data indicated the possible amphipathic α-helical conformation of the selected CPPs: (A) EASKKPRQKR peptide containing tumor penetrating motif (RXXR); (B) GIEFLKRGDK peptide containing tumor homing motif (RGD); (C) RSGARSKQRR peptide with +5.00 net charge; (D) VVLKKLKKSL peptide with high half-life (~100 hour) in mammalian cells; (E) SSRSRNSSRN peptide with the highest Boman index (~7.5); (F) MCYKRNRATR peptide containing tumor penetrating motif (RXXR). The structural motifs were shown as hydrophobic (yellow) and cationic (blue). Arrow illustrates direction of the hydrophobic moment (μH).

The 3D spatial shapes of CPPs were predicted by PEP-FOLD3 web server (**Fig 2**). Also, the helical wheel projection of these short peptides were obtained via Heliquest web server as indicated in **Fig 3**. A peptide comprising at least five adjacent hydrophobic residues (such as Leu, Ile, Ala, Val, Pro, Met, Phe, Trp, and Tyr) illustrates a hydrophobic face on a helical wheel projection [46].

## Discussion

Vaccination is one of the most effective strategies for control of dangerous pathogens. A potent vaccine must stimulate strong humoral and cellular immune responses in host [56]. The vaccine efficacy relies on various factors including the selected antigen, adjuvant and delivery system [57]. Therefore, many researchers have focused on development of novel and powerful delivery systems [9,58–60]. Since the discovery of CPPs, these short peptides were considered as a significant delivery system to enter diverse types of cargoes into cells due to their high cellular uptake efficiency. Several viruses such as HIV-1, Influenza A virus subtype H5N1, Dengue virus and HSV-1 contain CPPs in their proteome [11,30,34,61].

The bioinformatics strategies take scientists one step forward in screening and evaluating CPPs. Hence, the current study was planned to screen and identify novel and potent CPPs in the proteome of SARS-CoV-2 using *in silico* tools. To achieve this aim, we extracted the sequences of S, M, N, E, ORF1ab, ORF3a, ORF6, ORF7a, ORF8, and ORF10 proteins and submitted to CellPPD web server. The CellPPD is a support vector machine (SVM)-based prediction approach which was established to predict highly efficient cell penetrating peptides. The CellPPD method was based on binary profile of peptides that settle the information of both composition and order of residues in peptides [40]. The output of analysis using CellPPD web server was a large number of CPPs which subjected to several web servers for further analysis such as their physicochemical properties, uptake efficiency, toxicity, allergenicity, cellular localization, tendency for binding to plasma membrane, and prediction of 3D structure. Our results showed that the proteome of SARS-CoV-2 contains a large number of cell penetrating peptides. Most of the predicted CPPs were originated from Orf1ab polyprotein. Orf1ab polyprotein forms about two thirds of the SARS-CoV-2 genome that is translated into two polypeptides such as pp1a and pp1b. Next, these two polypeptides are processed and cleaved into sixteen non-structural proteins (nsp). Non-structural proteins possess crucial functions in the replication, transcription and pathogenesis of viral RNA [29]. Despite Orf1ab polyprotein, our data indicated that no CPP was found in the E protein. This protein is responsible for virus production and maturation [28]. Herein, twenty-four CPPs were predicted in spike (S) protein, as well. Furthermore, most of the predicted CPPs in S protein are amphipathic in nature. On the other hand, most of the predicted CPPs showed high uptake efficiency using *in silico* approaches. The studies demonstrated that several factors affect the uptake efficiency such as the number of arginine, the existence of tryptophan and its affinity to form helical structure, and orientation of tryptophan and arginine around the helix [62–64]. In addition, it should be considered that CPPs because of their natural pore-forming propensity and high hydrophobic moment (μH) could damage or destabilize the lipid bilayers irreversibly and so they showed cytotoxic effects. Hence, minimizing μH should be performed to reduce the membrane-disturbing by CPPs [65,66]. Our data indicated that most of the CPPs predicted from the proteome of SARS-CoV-2 were not toxic and allergen, and had appropriate half-life, as well as they could bind to plasma membrane with high potential and subsequently penetrate into cells. For example, Kajiwara *et al*. showed that H5N1 highly pathogenic avian influenza virus (HPAIV) infects host cells by recruiting CPP activity of the C-terminal domain of HA1 protein (HA314-346) [61]. Moreover, the N-terminal tail of capsid protein (CaP) from the plant-infecting brome mosaic virus (BMV) containing the arginine-rich motif was essential for penetration through cellular membranes [67]. Thus, it is possible that CPPs found in the SARS-CoV-2 proteome possess the potency for virus penetration into host cells.

On the other hand, CPPs are not cell-specific and thus they are internalized in most of the cell types through receptor-independent approach. Hence, to determine CPPs that might be cancer-specific or might enter cancerous cells effectively, the peptide sequence should possess the tumor homing motif (RGD) and/or tumor penetrating motif (RXXR). Moreover, the peptides harboring RXXR motif at their C-terminal region could enter tumor cells through binding to neuropilin receptor which was commonly expressed at the surface of tumor cells [19]. In our study, one of the SARS-CoV-2-derived CPPs (*i*.*e*., GIEFLKRGDK) contains RGD motif. This CPP with +1.00 net charge was soluble in water, non-toxic, and its half-life was about 30 hours in mammalian. Its cellular localization was predicted using TMHMM server. Interestingly, two CPPs such as EASKKPRQKR and MCYKRNRATR peptides included RXXR motif at their C-terminal regions. In detail, EASKKPRQKR peptide had +4.00 net charge and good water solubility. This peptide was non-toxic and non-allergen with about one hour half-life in mammalian. Also, the Boman index was 6.04 for this CPP (*i*.*e*., the values higher than 2.48 kcal/mol showed high binding potential), and its cellular localization was confirmed by TMHMM web server. Moreover, MCYKRNRATR peptide had +4.00 net charge and good water solubility. But this CPP was predicted as a toxic and allergen peptide with the estimated Boman index about 5.42. Additionally, TMHMM web server predicted its localization inside the cell. Therefore, based on our data, the efficiency of GIEFLKRGDK and EASKKPRQKR peptides can be further evaluated *in vitro* and *in vivo* as a delivery system in cancer therapy.

In the present study, only CPPs with 10 residues in length were predicted. As known, CPPs contain 5–50 residues in length [11]. Thus, we can design novel CPPs with more length and higher efficiency by addition of some sequences for delivery of different cargoes. For instance, we can add a hydrophilic lysine-rich domain derived from NLS of SV40 large T-antigen (KKKRKV) and a spacer domain (WSQP) to improve the efficiency of CPPs in DNA delivery as used in other studies [33]. In this study, as an example, by merging 11 overlapped CPPs derived from N protein such as KKSAAEASKK, KSAAEASKKP, SAAEASKKPR, AAEASKKPRQ, AEASKKPRQK, EASKKPRQKR, ASKKPRQKRT, SKKPRQKRTA, KKPRQKRTAT, KPRQKRTATK, and PRQKRTATKA peptides (with net charges of +4.00 and +5.00), a novel CPP was designed with 21 residues in length (*i*.*e*., KKSAAEASKKPRQKRTATKAY). This CPP had +7.00 net charge and good water solubility. Moreover, it was non-allergen and non-toxic with immunogenicity score about -0.70123 and D factor about 2.46 which would be located into cells as predicted by TMHMM web server. Surprisingly, when the SV40 large T-antigen NLS sequence and a spacer domain were conjugated to this CPP, we had a new CPP with 31 amino acids in length (*i*.*e*., KKSAAEASKKPRQKRTATKAYWSQPKKKRKV), +12.00 net charge, and good water solubility. This peptide was non-allergen and non-toxic with immunogenicity score about -1.49065 and D factor about 4.11, which was localized into cells as predicted by TMHMM web server. Indeed, using the conjugation of NLS and spacer to the designed CPP, the net charge and the probability of cellular localization inside cells were enhanced. Our predicted and designed CPP is similar to MPG CPP (27 residues in length, and +4.00 net charge) composed of peptide derived from HIV-1 glycoprotein 41, SV40 NLS and spacer domain. The MPG peptide was reported for delivery of DNA-based vaccine both *in vitro* and *in vivo* [33,68,69].

## Conclusion

In conclusion, novel and potent CPPs derived from the proteome of SARS-CoV-2 were identified using *in silico* methods. It is possible for relationship between these CPPs and rapid spreading the virus in host. Moreover, we designed a long and novel CPP conjugated to SV40 NLS and spacer domain that had high binding ability to membrane and localization inside cells. The designed CPP was similar to MPG CPP. This CPP can be further evaluated for DNA delivery *in vitro* and *in vivo* in future. Generally, the predicted and designed CPPs derived from the proteome of SARS-CoV-2 with different properties can be applied to deliver different cargoes in vaccine and drug development.

## Supporting information

S1 Fig The flowchart of overall study plan. (TIF)
