# Supplementary figures and images for: Exploring novel and potent cell penetrating peptides in the proteome of SARS-COV-2 using bioinformatics approaches
Source: PLoS One. 2021 Feb 19;16(2):e0247396. doi: 10.1371/journal.pone.0247396 (PMC7894964; doi:10.1371/journal.pone.0247396)

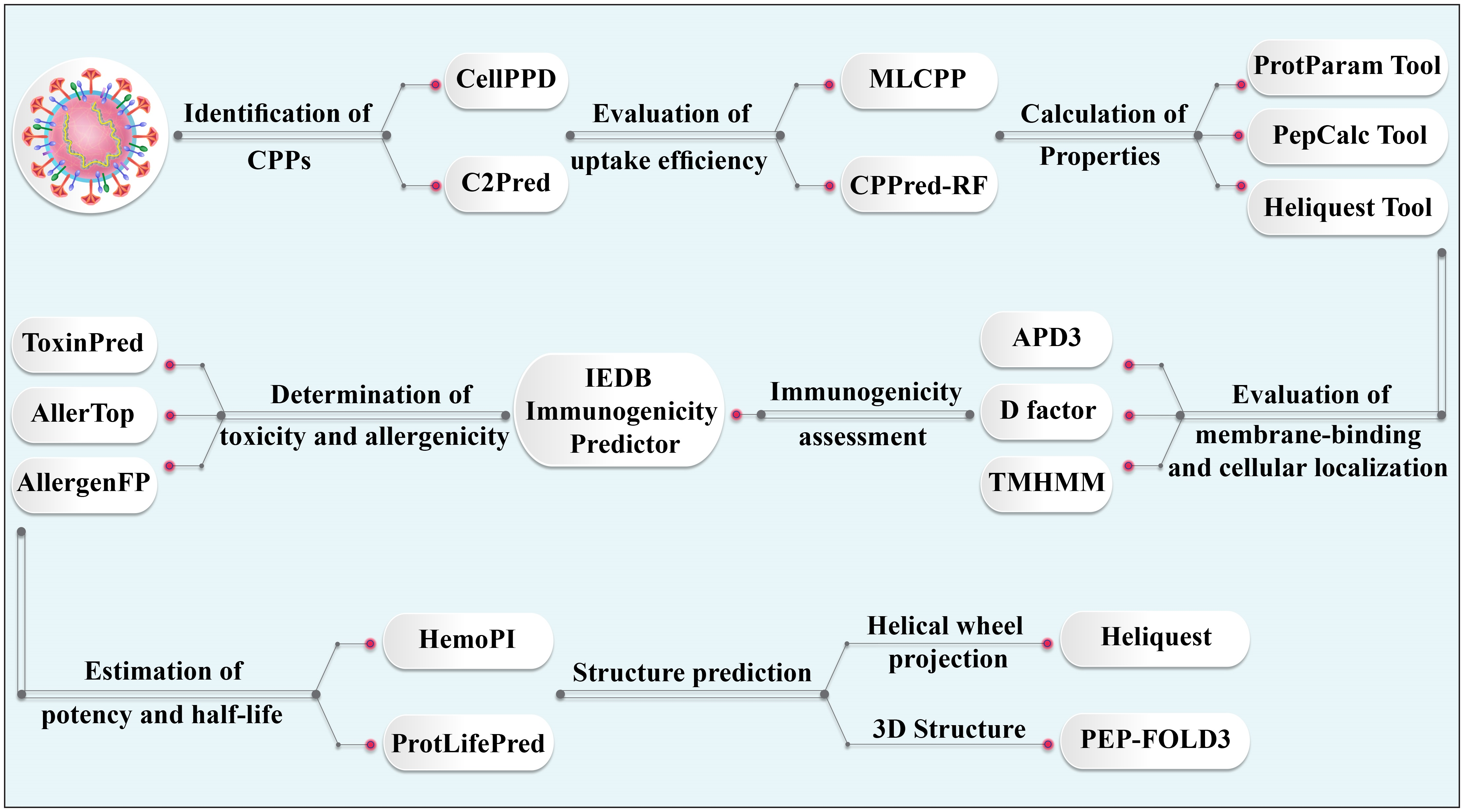

Supplement: S1 Fig — (TIF) [file pone.0247396.s001.tif]
